# Drugging the lncRNA MALAT1 via LNA gapmeR ASO inhibits gene expression of proteasome subunits and triggers anti-multiple myeloma activity

**DOI:** 10.1038/s41375-018-0067-3

**Published:** 2018-02-22

**Authors:** Nicola Amodio, Maria Angelica Stamato, Giada Juli, Eugenio Morelli, Mariateresa Fulciniti, Martina Manzoni, Elisa Taiana, Luca Agnelli, Maria Eugenia Gallo Cantafio, Enrica Romeo, Lavinia Raimondi, Daniele Caracciolo, Valeria Zuccalà, Marco Rossi, Antonino Neri, Nikhil C. Munshi, Pierosandro Tagliaferri, Pierfrancesco Tassone

**Affiliations:** 1 Department of Experimental and Clinical Medicine, Magna Graecia University of Catanzaro, Catanzaro, Italy; 2 Jerome Lipper Multiple Myeloma Center, Department of Medical Oncology, Dana-Farber Cancer Institute, Boston, MA USA; 3 Department of Oncology and Hemato-oncology, University of Milan, Milan, Italy; 4 Hematology Unit, Fondazione IRCCS Cà Granda, Ospedale Maggiore Policlinico, Milan, Italy; 5 Laboratory of Tissue Engineering, Rizzoli Orthopedic Institute, Palermo, Italy; 6 Pathology Unit, Pugliese-Ciaccio Hospital, Catanzaro, Italy; 7 VA Boston Healthcare System, West Roxbury, Boston, MA USA; 8 Sbarro Institute for Cancer Research and Molecular Medicine, Center for Biotechnology, College of Science and Technology, Temple University, Philadelphia, PA USA

## Abstract

The biological role and therapeutic potential of long non-coding RNAs (lncRNAs) in multiple myeloma (MM) are still to be investigated. Here, we studied the functional significance and the druggability of the oncogenic lncRNA MALAT1 in MM. Targeting MALAT1 by novel LNA-gapmeR antisense oligonucleotide antagonized MM cell proliferation and triggered apoptosis both in vitro and in vivo in a murine xenograft model of human MM. Of note, antagonism of MALAT1 downmodulated the two major transcriptional activators of proteasome subunit genes, namely NRF1 and NRF2, and resulted in reduced trypsin, chymotrypsin and caspase-like proteasome activities and in accumulation of polyubiquitinated proteins. NRF1 and NRF2 decrease upon MALAT1 targeting was due to transcriptional activation of their negative regulator KEAP1, and resulted in reduced expression of anti-oxidant genes and increased ROS levels. In turn, NRF1 promoted MALAT1 expression thus establishing a positive feedback loop. Our findings demonstrate a crucial role of MALAT1 in the regulation of the proteasome machinery, and provide proof-of-concept that its targeting is a novel powerful option for the treatment of MM.

## Introduction

Multiple myeloma (MM) is a B-cell malignancy characterized by abnormal proliferation of plasma cells (PCs) within the bone marrow (BM). Despite increased knowledge on the pathobiology of MM and the approval of new therapeutics, MM is still an incurable disease with a 5 years overall survival rate of about 45% [1].

Over the past decade, functional genomics and epigenomics studies have demonstrated that, similar to protein-coding genes, short non-coding RNAs are dysregulated and play key roles in the pathogenesis of human cancers [2, 3], including MM [4]. Notably, over half of the human genome is transcribed as long non-coding RNAs (lncRNAs), this term referring to non-coding transcripts longer than 200 nucleotides [5]. LncRNAs regulate gene transcription and mRNA translation by different mechanisms, including interaction with RNA-binding proteins, epigenetic modification of gene expression, or microRNA modulation [6]. Therefore, it is not surprising that lncRNAs are implicated in relevant biological processes such as development, differentiation, apoptosis, and cell cycle, and are dysregulated in cancer [7]. Consistently, it is becoming clear that lncRNAs may act as oncogenes or tumor suppressors [8], and may represent potential druggable targets [9].

Dysregulation of lncRNAs in the hematopoietic compartment has been proven to contribute to the onset of malignancies [10].

The metastasis-associated lung adenocarcinoma transcript 1 (MALAT1) is an evolutionarily conserved lncRNA, that plays a critical role both in the maintenance of the undifferentiated status of hematopoietic stem cells [11] and in B-cell activation [12]. By expression profiling of lncRNAs in PC dyscrasias, we previously demonstrated high expression of MALAT1 associated with onset of the disease and progression from normal PCs to overt MM [13]. MALAT1 has been found overexpressed in a wide variety of other hematological malignancies and solid tumors [14]. The high expression of MALAT1 in MM, along with its involvement in well-established cancer-associated pathways [14, 15], points to a role of this lncRNA in MM pathogenesis.

Here we characterized the biological sequelae of MALAT1 in MM, and we reported the first evidence of its druggability by a novel LNA gapmeR antisense oligonucleotide (ASO), which could lead to significant therapeutic advances in MM and other tumors.

## Materials and methods

### Cell cultures, drugs, and ASOs/siRNAs

Detailed information is provided in Supplementary Methods.

### Microarray gene expression profiling

See Supplementary Methods for protocols.

### Cell viability, proliferation, and migration assays

Cell viability was analyzed by Cell Titer-Glo (CTG; Promega), and S-phase DNA synthesis by BrdU Cell Proliferation assay (Cell Signaling Technology). For colony-forming assay, 200 cells were plated in triplicate in 1 ml of mixture composed of 1.1% methylcellulose (MethoCult^TM^ STEMCELL Technologies) in RPMI-1640 + 10% FBS. Crystal violet-stained colonies were scored after 2 weeks under an inverted microscope (Leica DM IL LED) at ×5 magnification. Cell migration was analyzed by Transwell migration assay (BD Biosciences) as described [16].

### Luciferase assays

For proteasome, ARE-Reporter, and ROS Luciferase Assays see Supplementary Methods.

### Plasmids, transfection, and transduction of MM cells

Plasmids used and related procedures are reported in Supplementary Methods.

### Quantitative real-time PCR

RNA extraction and quantitative real-time PCR (qRT-PCR) were performed as described [17]. Procedures are in Supplementary Methods.

### Western blot and antibodies

Whole cell protein extracts were prepared using NP40 containing Halt Protease Inhibitor cocktail (Invitrogen, Thermo Scientific). Western blot (WB) was performed as reported [18] (Supplementary Methods).

### Chromatin and RNA immunoprecipitation

For chromatin immunoprecipitation (ChIP) experiments, the Pierce Agarose ChIP Assay Kit (Thermo Fisher Scientific) was used. RNA immunoprecipitation (RIP) was performed using Imprint® RNA Immunoprecipitation Kit (Sigma Aldrich); see Supplementary Methods for protocols.

### In vivo study

The MM xenograft model used is described in Supplementary Methods.

### Immunohistochemistry and immunofluorescence

See Supplementary Methods.

### Statistical analysis

Each experiment was performed at least three times, and values were reported as mean ± SD. Data were analyzed using Student's *t* tests for two group comparisons or a one-way analysis of variance (ANOVA) for multiple comparisons using the Graphpad software (GraphPad Software, La Jolla, CA, USA). *P*-value < 0.05 was considered significant.

## Results

### MALAT1 regulates growth and survival in MM cell lines and primary cells

We first interrogated three microarray datasets and observed increasing expression of MALAT1 in PCs from MGUS, SMM and overt MM as compared to normal PCs (Supplementary Fig. S1a). To evaluate the potential prognostic value of MALAT1 in MM, we analyzed both a clinically annotated proprietary dataset and the large TT2/TT3 trials cohort from the University of Arkansas encompassing more than 550 patients. However, we did not find any significant correlation between MALAT1 expression and overall survival or time to relapse (Supplementary Fig. S1b-c).

**Fig. 1:**
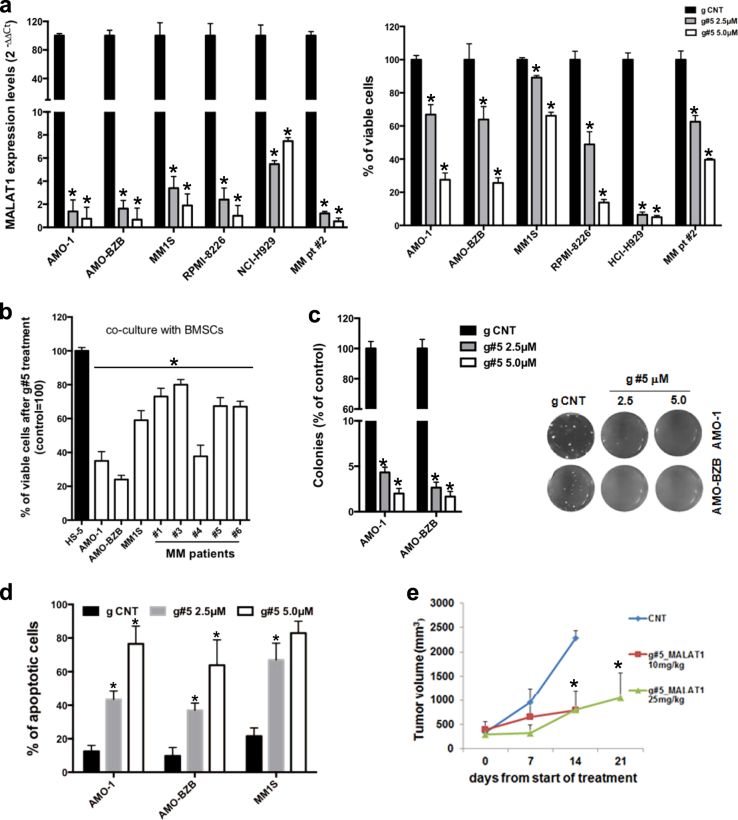
Effects of MALAT1 manipulation on in vitro and in vivo MM growth. **a** MALAT1 relative levels (left panel) and cell viability of MM cells (right panel) were determined by qRT-PCR and CTG assay respectively, 4 days after treatment with 5 μM g CNT or g#5. **b** CTG assay in MM cell lines and primary PCs co-cultured on HS-5 stromal cells, and treated for 4 days with 5 μM g CNT or g#5. **c** Colony formation assay performed on MM cell lines treated for 14 days with 5 μM g CNT or g#5; representative pictures of colonies at day 14 are also shown. **d** Annexin V staining of MM cell lines, 5 days after treatment with naked g CNT or g#5. Data are representative of at least three independent experiments. **e** Caliper measurements of tumor volumes in mice bearing AMO-ABZB-luc xenografts IP-treated with g CNT (25 mg/kg) or g#5. *p < 0.05

To investigate the role of MALAT1 in MM, we exploited both gain and loss of function approaches. Ectopic expression of MALAT1 increased viability, proliferation, and migration of low MALAT1 expressing AMO-1 cells (Supplementary Fig. S1d), and the same effects were observed in bortezomib-resistant AMO-BZB cells (Supplementary Fig. S2a-c). MALAT1-dependent proliferative advantage was supported by activation of oncogenic pathways, as demonstrated by the increase in phosphorylated AKT, p65-NF-κB, ERK1/2, and CREB (Supplementary Fig. S2d). To knock-down (KD) MALAT1, we used novel LNA-gapmeR ASOs, that trigger RNAse-H-dependent degradation of lncRNAs [19]. Transfection of two different LNA-gapmeRs targeting MALAT1 (henceforth referred to as g#5 and g#9) significantly reduced MALAT1 expression, and this effect translated into reduced viability and migration of tumor cells (Supplementary Fig. S3a-c). Transfection of MALAT1-targeting siRNAs also reduced MM cell viability (Supplementary Fig. S3d), although to a lesser extent than LNA-gapmeRs. At micromolar concentration, naked LNA-gapmeRs passively cross the plasma membrane (gymnosis) [20]. We thus evaluated the sensitivity to naked g#5 of human MM cell lines and primary patient CD138^+^ MM cells. After 96 h of exposure to g#5, a potent downregulation of MALAT1 was observed, with a parallel decrease in cell viability (Fig. 1a); conversely, PBMCs from healthy donors were not sensitive to g#5 (Supplementary Fig. S3e), thus suggesting a favorable therapeutic index. Importantly, exogenous MALAT1 expression completely rescued the effects of g#5 on cell viability (Fig. S3f). We next evaluated g#5 effects in the context of the BM *milieu* [21]: treatment with g#5 suppressed the viability of MM cell lines or patient-derived MM cells co-cultured on HS-5 cells, without any effect on HS-5 viability (Fig. 1b; MALAT1 expression in patient MM cells is reported in Supplementary Fig. S3g). Moreover, exogenous cytokines did not counteract g#5 effect on cell viability (Supplementary Fig. S3h). G#5 treatment dramatically suppressed the clonogenicity of MM cells (Fig. 1c), while MALAT1 overexpression increased colony formation (Supplementary Fig. S3i).

### Caspase-dependent induction of MM cell death by MALAT1 inhibition in vitro and in vivo

To uncover the biological scenario underlying cytotoxicity of MALAT1 inhibition, we analyzed the effects of g#5 treatment on cell cycle and apoptosis. After 72 h, g#5 altered cell cycle profile, with a remarkable inhibition of S-phase (Supplementary Fig. S3l), which was confirmed by reduced BrdU uptake (Supplementary Fig. S3m). Prolonged exposure of MM cells to g#5 up to 5 days triggered apoptotic cell death (Fig. 1d); both intrinsic and extrinsic apoptotic pathways were induced in MM cell lines and primary patient CD138^+^ MM cells by g#5, as assessed by WB (Supplementary Fig. S3n). Exposure to pan-caspase inhibitor zVAD-FMK reverted g#5-inhibitory effects on cell viability (Supplementary Fig. S3o).

Finally, we analyzed the effect of g#5 treatment in vivo, in a murine xenograft model of human MM. Treatment doses were selected based on in vitro activity of g#5 and on similar LNA ASOs [22]. Notably, g#5 reduced the growth of AMO-BZB-luc xenografts (Fig. 1e and Fig. S4a); on-target activity of g#5 in vivo was confirmed by reduced MALAT1 levels in resected tumor samples after g#5 treatment (Supplementary Fig. S4b). H&E staining of mice vital organs did not highlight any change in tissue architecture (Supplementary Fig. S4c); lack of toxicity was also supported by the absence of neurological changes or weight loss of treated animals (data not shown). Importantly, increase in caspase-3 on g#5-treated xenografts confirmed apoptosis induction (Supplementary Fig. S4d).

### Identification of proteasome-associated genes as targets of MALAT1 in MM

**Fig. 2:**
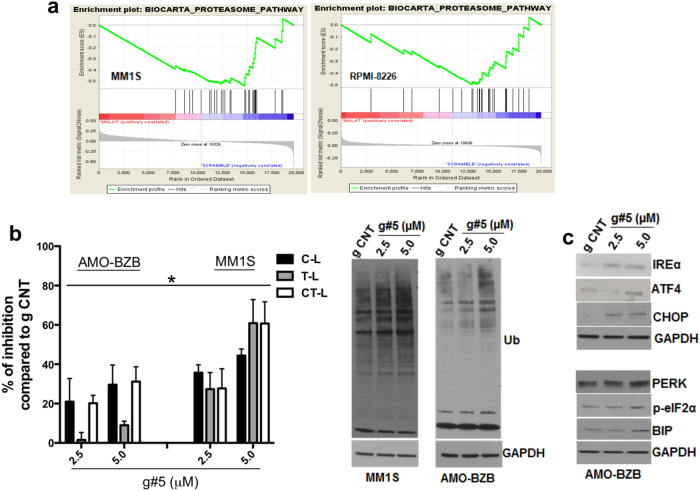
MALAT1 depletion reduces proteasome gene expression in MM cells. **a** GSEA performed 48 h after transfection with 50 nM g CNT or g#5 (nominal *p*-values for RPMI-8226 and MM1s were 0.00 and 0.028, respectively). **b** Caspase-like (C-L), Trypsin-like (T-L), Chimotrypsin-like (CT-L) activities evaluated by proteasome assay (Promega), 4 days after delivery of naked gCNT or g#5; WB of polyubiquitinated (Ub) proteins is reported on the right. **c** WB of ER stress markers 4 days after delivery of naked gCNT or g#5. * = *p* < 0.05

In order to identify MALAT1 targets unveiling its functional role, we carried out gene expression profiling in two MM cell lines after MALAT1 KD. GSEA identified the proteasome as the only pathway downregulated by g#5 (Fig. 2a). Using qRT-PCR and WB, we confirmed reduced expression of selected proteasome subunits as well as of proteasome maturation protein (POMP), responsible for assembly of β subunit rings [23], upon g#5 treatment (Supplementary Fig. S5a-b); similar results were obtained after transfection of MALAT1-targeting siRNAs (Supplementary Fig. S5c). Trypsine-like, chymotrypsin-like, and caspase-like proteasome activities were also significantly inhibited by g#5, resulting in accumulation of polyubiquitinated proteins (Fig. 2b); conversely, proteasome activities were augmented by MALAT1 overexpression (Supplementary Fig.S5d). These data suggest a role of MALAT1 in the proteasome pathway. To confirm in vitro data, we evaluated gene expression signatures associated with MALAT1 expression in primary MM cells. MM patients were divided in four groups according to MALAT1 expression. Functional enrichment analysis of genes differentially expressed between the two groups with high (I quartile) and low (IV quartile) MALAT1 (Supplementary Fig. S5e) showed significant enrichment of the proteasome pathway in high MALAT1 group (Supplementary Table S1), strengthening the role of MALAT1 in proteasome modulation.

Accumulation of polyubiquitinated proteins induces endoplasmic reticulum (ER) stress and triggers the unfolded protein response [24]. G#5 treatment increased phosphorylation of eIF2a, and induced ER stress sensor proteins such as inositol-requiring enzyme 1α (IRE1α), the protein kinase R (PRKR)-like ER kinase (PERK), the chaperone BiP/GRP78, the Activating Transcription Factor 4 (ATF4), and the UPR-induced proapoptotic CCAAT/enhancer-binding protein (C/EBP) homologous protein (CHOP) (Fig. 2c). These results suggest that ER stress-induced apoptosis may contribute to cytotoxicity of g#5.

### MALAT1 regulates NRF1/2 signaling pathway

**Fig. 3:**
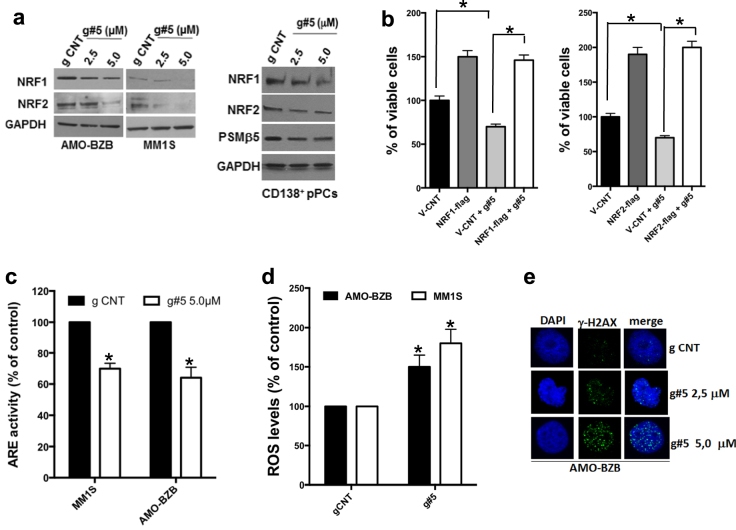
MALAT1 regulates NRF1 and NRF2 expression and activity. **a** WB of NRF1, NRF2, and PSMβ5 in MM cell lines or patient-derived MM cells, 5 days after delivery of naked g CNT or g#5. **b** CTG viability assay was performed in AMO-BZB cells transfected with 2.5 μg of NRF1-flag or NRF2-flag expression vectors, then treated for 4 days with 2.5 μM naked g#5. **c** Luciferase assay performed in cells transfected with an ARE reporter (Promega), and then treated for 4 days with 5.0 μM g CNT or naked g#5. **d** ROS measurement by luminescent assay, 4 days after delivery of 5.0 μM naked g CNT or g#5. **e** Representative immunofluorescence of γH2AX foci, 4 days after delivery of g CNT (5.0 μM) or g#5 (×63 magnification). Data are representative of at least three independent experiments. *p < 0.05

Our results indicate that inhibition of MALAT1 is associated with a decrease in both proteasome subunit mRNA levels and proteasome activity. NRF1 and NRF2 are key transcription factors that control proteasome gene expression in cells upon binding to Antioxidant Response Element (ARE)-bearing promoters [25–27]; accordingly, we observed reduced expression of proteasome-associated genes in MM cells transfected with NRF1 and NRF2 siRNAs (Supplementary Fig.S6a-b), and corresponding decrease of MM cell viability (Supplementary Fig.S6c). We hypothesized that NRF1 and NRF2 might be involved in MALAT1-dependent regulation of proteasome gene expression. WB indicated a significant impact of MALAT1 inhibition and/or overexpression on the protein levels of NRF1 and NRF2; importantly, downregulation of NRF1, NRF2, and PSMβ5 proteins was also confirmed in primary CD138^+^ MM cells treated ex vivo with g#5 (Fig. 3a and Fig. S6d). Ectopic NRF1 and NRF2 abrogated g#5-induced downregulation of PSMβ5 mRNA (Fig. S6e-f) and rescued the effects on MM cell viability (Fig. 3b), suggesting a significant role of NRF1/2 on the oncogenic potential of MALAT1 in MM.

NRF1 and NRF2 are involved in ROS detoxification by activating ARE-bearing anti-oxidant genes’ promoters [28]. Consistent with NRF1 and NRF2 downregulation, g#5 inhibited the activity of an ARE-driven reporter (Fig. 3c), downregulated mRNAs of antioxidant genes (Supplementary Fig.S7a), and enhanced ROS production (Fig. 3d). Elevated oxidative stress is present in cancer cells, and excessive ROS may promote DNA damage [29]. Of note, g#5 increased DNA damage of MM cells, evidenced by increased γH2AX phosphorylation (Supplementary Fig. S7b) and accumulation of γH2AX nuclear foci (Fig. 3e) in MALAT1-depleted cells. These data indicate that MALAT1 antagonism inhibits NRF1/2 expression and activity in MM cells.

### MALAT1 epigenetically regulates the NRF1/2-negative regulator KEAP1

**Fig. 4:**
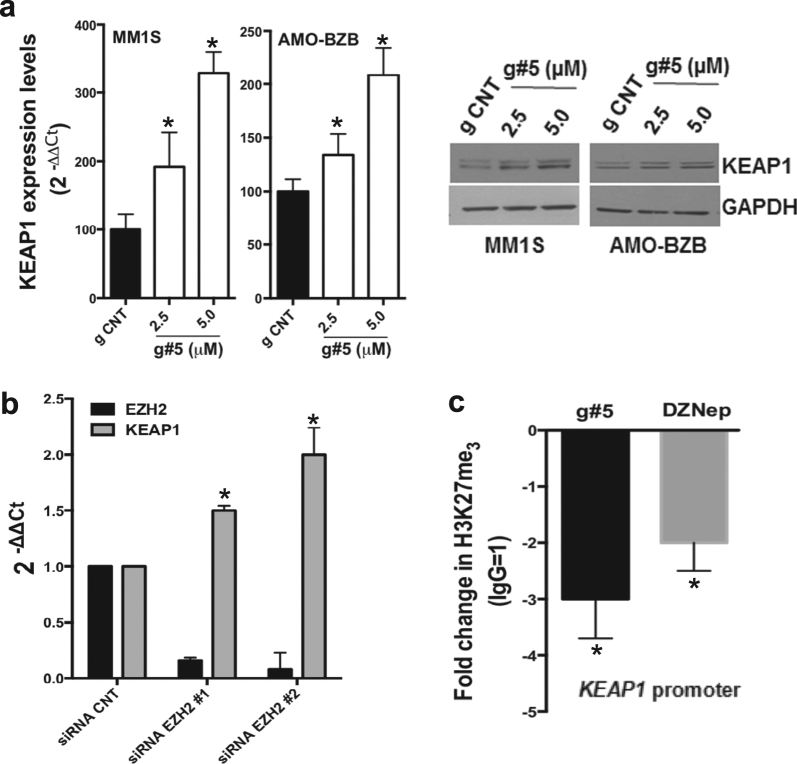
MALAT1 epigenetically regulates KEAP1. **a** qRT-PCR of KEAP1 mRNA relative expression, 5 days after delivery of naked g CNT or g#5; right panel shows WB of KEAP1. **b** qRT-PCR of KEAP1 and EZH2 mRNA levels in AMO-BZB cells, 24 h after transfection with 100 nM of the corresponding siRNAs. **c** qPCR for *KEAP1* promoter performed after ChIP with H3K27Me3 antibody, in AMO-BZB cells treated for 72 h with 2.5 μM naked g#5 or 2 μM DZNep. Data are representative of at least three independent experiments. *p < 0.05

NRF1/2 protein turn-over in tumor cells is controlled by the kelck-like EZH-associated protein 1 (KEAP1) and other ubiquitin ligase complexes [30, 31]. In AMO-BZB cells, transfection of KEAP1-targeting siRNAs upregulated both NRF1 and NRF2 proteins, as well as PSMβ5 subunit (Supplementary Fig. S8), thus supporting a negative role of KEAP1 on NRF1 and NRF2 stability in MM cells. Importantly, KEAP1 mRNA and protein levels resulted upregulated upon g#5 treatment (Fig. 4a), while reduced by ectopic MALAT1 (Supplementary Fig. S9). Since interaction of MALAT1 with EZH2 has been proven to negatively impact gene expression [32], we asked whether MALAT1 could epigenetically regulate KEAP1. Using a RIP assay, firstly we confirmed a physical interaction between EZH2 and MALAT1 in MM cells (Supplementary Fig. S10). Similar to g#5, genetic (via EZH2-siRNAs; Fig.4b) or pharmacological (via DZNep; Supplementary Fig.S11a) inhibition of EZH2 resulted in KEAP1 upregulation. Importantly, inhibition of either EZH2 or MALAT1 by DZNep and g#5 respectively, displaced H3K27me3 from the *KEAP1* promoter, as assessed by ChIP (Fig. 4c). Finally, ectopic expression of EZH2 abrogated g#5-induced upregulation of KEAP1 mRNA (Fig. S11b). Collectively, these results indicate that MALAT1 cooperates with EZH2 to repress KEAP1 transcription, and suggest that MALAT1 inhibition may target the proteasome in MM cells by upregulating KEAP1, which negatively impacts NRF1 and NRF2 expression.

### NRF1 and MALAT1 establish a feedback loop with clinical significance

**Fig. 5:**
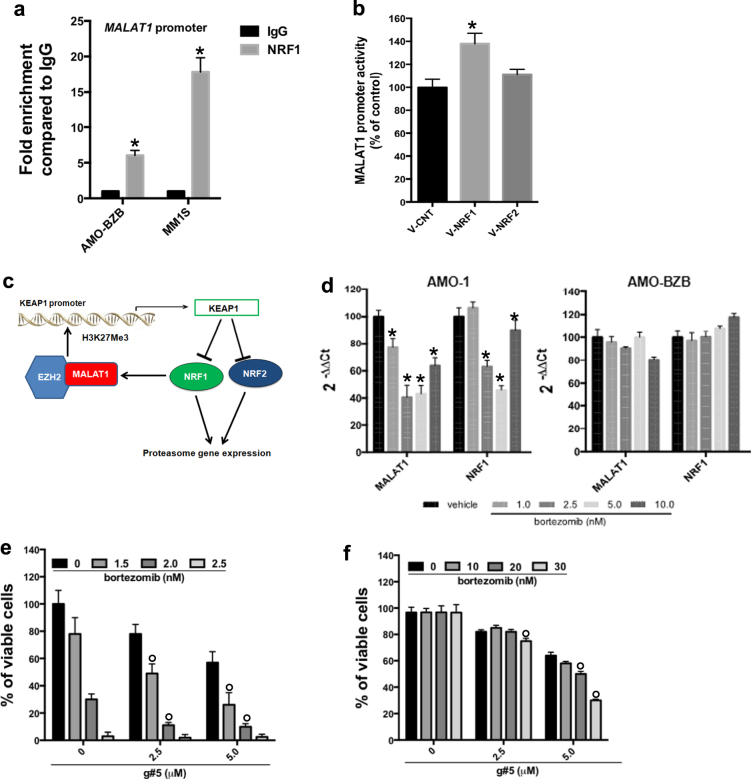
NRF1 induces MALAT1 expression in a clinically relevant feedback loop. **a** qPCR for *MALAT1* promoter performed after ChIP with an NRF1 antibody or IgG control. **b** Luciferase assay carried out in AMO-BZB cells, 48 h after transfection with MALAT1 promoter reporter (2.5 μg), together with 2.5 μg of NRF1 (V-NRF1), NRF2 (V-NRF2), or empty vector (V-CNT). **c** Cartoon illustrating the regulatory loop identified in this study. **d** qRT-PCR of MALAT1 and NRF1, 24 h after bortezomib treatment. CTG viability assay performed in AMO-1 cells (**e**) and in AMO-BZB cells (**f**), 4 days after treatment with g#5 and bortezomib. *p < 0.05; °combination index < 1.0 (calculated using Calcusyn)

In silico search of TF binding sites within MALAT1 promoter revealed a putative NRF1 consensus (Supplementary Fig. S12). Indeed, using a ChIP assay, we observed significant enrichment of NRF1 at *MALAT1* promoter (Fig. 5a); moreover, ectopic expression of NRF1, but not NRF2, transactivated *MALAT1* promoter (Fig. 5b), as shown by luciferase reporter assays. Finally, silencing of NRF1 resulted in MALAT1 downregulation in MM cells, while no effect was observed in NRF2-silenced cells (Fig. S13a). Collectively, these data support the mutual regulation of MALAT1 and NRF1 within a regulatory loop ultimately impacting the proteasome machinery (Fig. 5c). To evaluate the clinical significance of this loop, firstly we analyzed NRF1 and MALAT1 expression in isogenic AMO-1 cell lines treated with bortezomib. Interestingly, bortezomib downregulated MALAT1 and NRF1 only in AMO-1 bortezomib-sensitive (Fig. 5d; Fig.S13b), indicating that MALAT1/NRF1 loop is overactivated in drug-resistant cells. To decipher the role of MALAT1 in bortezomib sensitivity, we combined g#5 with bortezomib and evaluated effects on cell viability: notably, g#5 enhanced bortezomib activity on AMO-1 (Fig. 5e) and on MM patient-derived primary PCs (Fig. S13c), and overcame bortezomib resistance in AMO-BZB cells (Fig. 5f). These data indicate that g#5 disrupts MALAT1/NRF1 loop and its combination with bortezomib triggers synergistic cytotoxicity.

## Discussion

An unprecedented boom of research has recently demonstrated dysregulation of short ncRNAs in MM, and their potential as therapeutic targets [2, 4]; conversely, the role of lncRNAs in MM pathobiology remains to be elucidated.

In this study, we investigated and functionally characterized MALAT1, a nuclear-localized lncRNA highly expressed in MM [13]. The tumor-promoting role of MALAT1 has been demonstrated in a variety of solid malignancies, where it modulates essential pathways promoting proliferation, escape from apoptosis, migration and invasion [15].

Importantly, we observed MALAT1 progressively upregulated in PCs from MGUS, SMM and overt MM patients as compared to normal PCs, suggesting a potential role in MM pathogenesis; however, we failed to demonstrate any significant association between MALAT1 expression and the clinical outcome in two available clinically annotated datasets tested.

We also performed a comprehensive analysis of the molecular perturbations produced by MALAT1 in MM cells, providing the first evidence of its potential as therapeutic target in MM. To efficiently target MALAT1, we used a novel LNA gapmeR ASO, that represents a unique tool to KD lncRNAs, even more efficiently than RNA interference [33]. LNA gapmeR ASOs have a central DNA gap that binds the RNA target, and triggers its RNase H-dependent degradation; the presence of phosphorothioate confers nuclease resistance in body fluids [34], while LNA increases affinity to the target [19]. ASOs are nowadays becoming an attractive therapeutic modality to target undruggable pathways [35]. In this light, we here demonstrated that gymnotic delivery of MALAT1-targeting 16mer LNA gapmeR g#5 is a potent anti-MM agent that decreases cell proliferation even in the presence of the BM *milieu*, and triggers apoptosis in both MM cell lines and patient-derived PCs. The translational relevance of our findings is underscored by significant anti-tumor activity of g#5 in a humanized murine model of MM, along with optimal tumor uptake and no evidence of systemic toxicity.

By transcriptome analysis, we found that g#5 downmodulated proteasome gene expression, and enrichment of proteasome pathway genes was observed in vivo in high MALAT1 MM patients. The proteasome is a multi-catalytic proteinase complex, responsible for degradation of damaged and misfolded ubiquitinated proteins [36]. Proteasome inhibition prefentially kills malignant cells, and represents the most clinically relevant target therapy in MM [37]. Intriguingly, g#5 treatment led to inhibition of all three proteasome catalytic activities, along with accumulation of polyubiquitinated proteins. Mechanistically, MALAT1-dependent proteasome regulation occurred via the NRF1-2/KEAP1 pathway. NRF1 and NRF2 are known transcriptional activators of proteasome genes [25–27]. As in other cancer types [15, 32], MALAT1 was found to interact with the methyltransferase EZH2 also in MM cells, and both MALAT1 and EZH2 inhibitors reduced H3K27Me3 at *KEAP1* promoter, thus upregulating KEAP1 mRNA. As a consequence, inhibition of MALAT1 decreased the expression and activity of KEAP1 targets NRF1 and NRF2.

Finally, NRF1 was found to bind to and transactivate *MALAT1* promoter, providing evidence of a novel regulatory loop in MM cells, whose targeting by g#5 enhanced bortezomib anti-tumor activity both in drug sensitive and resistant MM cells.

MALAT1 targeting may therefore provide a novel strategy to simultaneously block all the proteasome catalytic activities. It is tempting to speculate that g#5 might confer therapeutic advantage when compensatory hyperactivation of trypsine and caspase-like activities emerge as a consequence of bortezomib resistance [38].

In conclusion, our study sheds light on a novel MALAT1-dependent regulation of MM proteasome machinery, and provides the first pre-clinical demonstration of a unique effective MALAT1-targeting ASO as new powerful therapeutic agent for MM treatment.

## Electronic supplementary material

Supplementary Materials and Methods

Supplementary Figures and Tables

Legends to Supplementary Figures and Tables
