# Supplementary material for: Drugging the lncRNA MALAT1 via LNA gapmeR ASO inhibits gene expression of proteasome subunits and triggers anti-multiple myeloma activity
Source: Leukemia. 2018 Feb 22;32(9):1948–57. doi: 10.1038/s41375-018-0067-3 (PMC6127082; doi:10.1038/s41375-018-0067-3)
Supplement: Supplementary file 1 — Supplementary Materials and Methods [file 41375_2018_67_MOESM1_ESM.pdf]

Supplementary Methods

Cell lines and cultures, drugs and oligonucleotides

MM cell lines NCI-H929, RPMI-8226, U266 SKMM1, MM1s and JJN3 were purchased from DSMZ, which certified authentication performed by short tandem repeat DNA typing; the bone marrow stromal cell line HS-5 were purchased from the American Type Culture Collection (Rockville, MD, USA); KMS11 cell line was obtained by Japanese Collection of Research Bioresources (National Institute of Health Sciences Japan). AMO-1 and AMO-BZB cells were kindly provided by Dr. C. Driessen (University of Tubingen, Germany); AMO-BZB were not further authenticated, but confirmed to have the described drug-resistant phenotype. All these cell lines were immediately frozen and used from the original stock within 6 months. Human MM cell lines were cultured in RPMI/1640 media containing 10% FBS (GIBCO; Life Technologies, Carlsbad, CA), 2 µmol/L glutamine, 100 U/mL penicillin, and 100 µg/mL streptomycin (GIBCO; Life Technologies, Carlsbad, CA) and tested for mycoplasma contamination. Peripheral blood mononuclear cells (PBMCs) and $CD138^{+}$ cells from BM of MM patients were isolated by Ficoll-hypaque (Lonza Group, Basel, Switzerland), followed by anti-CD138 microbeads (Milteny Biotech, Gladbach, Germany) selection, in accordance with the Declaration of Helsinki following informed consent and Institutional Review Board (University of Catanzaro, Italy) approval. Primary cells were then cultured in RPMI with 20% FBS supplemented with IL-6 (2.5 ng/ml).

Bortezomib and 3-Deazaneplanocin A (DZNep) were from Selleck Chemicals LLC (Munich, Germany) and Sigma Aldrich (Sigma, Steinheim, Germany), respectively. LNA™ longRNA gapmeRs hsa-malat1_5 (named g#5) and hsa-malat1_9 (named g#9) were from Exiqon (Vedbaek, Rudersdal, Denmark); their sequence was: ACATTGCCTCTTCATT (g#5) and AGATTCCGTAACTTTA (g#9); as control, a LNA™ longRNA gapmeR negative control (named g CNT: GCTCCCTTCAATCCAA) was used. Silencer® Select siRNA for NRF1 (siNRF1, cat#129900, HSS107126, HSS107127), for NRF2 (siNRF2, cat#129900, HSS181505, HSS181506), or control (siCNT, cat #AM4611) were from Thermo Fisher Scientific; siRNAs targeting KEAP1(SignalSilence® KEAP1 siRNA, cat#5285S) were from Cell Signaling Technology (Danvers, MA, USA). Lincode MALAT1 SMARTpool siRNA (cat#R-18797800) and ON-Target plus Non-targeting siRNA#1 negative control were from Dharmacon GE Healthcare (Lafayette, CO).

Microarray Gene Expression Profiling

GEP was obtained after cell transfection with g#5 or g CNT in 3 parallel experiments. RNA was extracted by RNeasy Mini kit (Qiagen, Hilden, Germany). A total of 300ng of RNA was used as starting material for preparing the hybridization target by using the GeneChip® WT PLUS Reagent Kit (Affymetrix Inc., Santa Clara, CA, USA). The integrity, quality and quantity of tRNA were assessed by the Agilent Bioanalyzer 2100 (Agilent Technologies, Santa Clara, CA, USA) and NanoDrop 1000 Spectrophotometer (Thermo Scientific, Wilmington, DE). The amplification of cRNA, the clean-up and the fragmentation were performed according to the Affymetrix's procedures. Microarray data were generated by GeneChip® Human Transcriptome 1.0 Array (Affymetrix Inc.). Arrays were scanned with an Affymetrix GeneChip Scanner 3000. Raw data produced by the Affymetrix Platform (i.e. CEL files) were first processed using Affymetrix Expression Console (EC). Pre-processing phase was performed according to Affymetrix guidelines and micro-CS software. Raw data were normalized using probe logarithmic intensity error (PLIER) algorithm coupled to quantile normalization. Annotation of data was also performed using Affymetrix Provided Libraries and EC version 1.4.1. Differential expression was assessed using a linear model method. *P*-values were adjusted for multiple testing using the Benjamini and Hochberg method. Tests were considered to be significant for adjusted $P<0.05$. Data are available through GEO accession number GSE108824.

Functional characterization of MALAT1 was performed using the GSEA software v.2.2.1 and the gene sets from Hallmarks, Kegg and Reactome collections. Ranking of coding genes from genes sets used the lncRNAs expression as a continuous phenotype and the Pearson's correlation as metric. Gene sets were considered significant if the false discovery rate was $\leqslant$ 0.25 under 1000 permutations. The 1093 differentially expressed genes between the two groups with high (I quartile) and low (IV quartile) MALAT1 were obtained by Significant Analysis of Microarrays v5.00, using the tool provided for the shiny package in R software (https://github.com/MikeJSeo/SAM), as described (1). The cutoff point for statistical signifcance (at a q-value 0) was determined by tuning the Δ parameter on the false discovery rate and controlling the q-value of the selected probes.

Antibodies for Western Blotting

The following antibodies were from Cell Signaling: anti-CREB(#9197), -phosphoCREB Ser133 (#9198), -NF-κB p65 (#8242), -phospho-NF-κB p65 Ser536 (#3033), -AKT(#9272), -phospho-AKT Ser473 (#4060), -phospho-p44/42 MAPK (Erk1/2) (Thr202/Tyr204, #4370), - p44/42 MAPK (Erk1/2, #9107), -PARP (#9532), -PSMβ5 (#12919), -Ubiquitin (#3936), -CHOP (#2895), -ATF-4 (#11815), -IREα (#3294), -PERK (#5683), -Phospho-eIF2α (#3398), -BIP (#3177), -Caspase-8 (#9746), -Caspase-3 (#9665), -KEAP1 (#8047), -NRF2 (#12721), -EZH2 (#5246), -POMP (#15141), and -phospho-Histone H2A.X Ser139 (#9718). Anti-PSMβ4 (sc-100454) and -GAPDH (sc-25778) were from Santa Cruz Biotechnology (Dallas, TX, USA).

In vivo study

Female SCID/NOD mice (6- to 8-weeks old; Harlan Laboratories, Inc., Indianapolis) were housed and monitored in our Animal Research Facility. All protocols were approved by our University Hospital Institutional Ethical Committee, and conducted according to the National Directorate of Veterinary Services (Italy). Mice were subcutaneously inoculated in the interscapular area with 5.0×10^6 luciferase-engineered AMO-BZB cells. When tumors became palpable, 5 mice/group were randomized to receive intraperitoneal treatments with either g#5 (10 or 25 mg/kg per mouse) or g CNT (25mg/kg), 2 days/week for a total of 5 injections. Randomization was performed in order to achieve a starting point with no significant differences between the experimental groups. Sample size (number of animals) was chosen based on our previous experience in the experimental treatment of MM xenografted NOD/SCID mice (2). Tumor sizes were measured in single blinded as previously described (2). Mice were sacrificed when their tumors reached 2 cm in diameter or in the event of major compromise in their quality of life. Tumor volume was measured by IVIS Lumina II System or by caliper measurement using the formula: $V= (a^2 \times b)/2$; where a is the width and b is the length of the tumor.

Proteasome activity assay

Proteasome activities were measured by Proteasome-Glo™ Assay (Promega), according to the manufacturer's instructions.

ARE Reporter Assay

MM cells were electroporated with the ARE (antioxidant response element) reporter using the Cignal Antioxidant Response Reporter kit (CCS-5020L, Qiagen). Firefly and Renilla luciferase activities were evaluated consecutively using the dual-luciferase assay kit (Promega).

ROS Assay

ROS levels were measured by ROS-Glo^{TM} H_2O_2 assay (Promega), according to the manufacturer's instructions.

Immunohistochemistry

Retrieved tumors and organs from treated mice were immersed in 4% buffered formaldehyde, and 24 hours later washed, dehydrated, and embedded in paraffin. Hematoxylin-eosin (H&E) staining was performed on 4 µm sections mounted on poly-lysine slides. Tissues sections were observed by using optical microscope OLYMPUS BX51 (Olympus Corporation, Tokyo, Japan). For immunohistochemistry staining, tumor and organs slices (2 µm size) were deparaffinized and pre-treated with the Epitope Retrieval Solution 2 (EDTA-buffer pH 8.8) at 98°C for 20 min. After washing steps, peroxidase blocking was carried out for 10 min using the Bond Polymer. All procedures were performed using the Benchmark XT-Automated Immunohistochemistry instrument (Ventana Medical Systems, Oro Valley, AZ, USA). Tissues were again washed and then incubated with the primary antibody directed caspase-3 (Novocastra, clone JHM62; 1:500) and Ki67 (Dako, clone: MIB-1; 1:150). Subsequently, tissues were incubated with polymer for 10 min and developed with DAB-Chromogen for 10 min. Slides were counterstained with hematoxylin for 12 min. The experiments were repeated at least three times.

Immunofluorescence

Cells were harvested, centrifuged onto glass slides (Cytospin 4, Thermo Scientific), then fixed in 4% paraformaldehyde in PBS1X for 12 min at 22°C, followed by three 5-min washes in PBS. Cells were permeabilized (0.1% Triton X-100 in PBS, 15-min), washed in PBS (3X, 5 min each), blocked 1 h at 22°C with 1.5% BSA in PBS, and then incubated O/N at 4°C with phospho-Histone H2A.X Ser139 antibody (Cell Signaling, 1:200, cat. #9718); thereafter, slides were washed in PBS (3X, 5 min each), and incubated 1 h at 22°C in the dark, with goat anti-rabbit Alexa Fluor 488 (Invitrogen 1:1000). After 3 PBS washes,

cells were mounted under coverslips with DAPI-containing Vectashield (Vector Laboratories). Images were acquired under an SP2 Leica Zeiss confocal laser-scanning microscope, with a 63X oil objective.

Flow cytometry

Phycoerythrin (PE)-conjugated CD138 mAb (CD138-PE; Imgenex, San Diego, CA) was used to evaluate surface expression of CD138. Annexin V/7-Aminoactinomycin (7-AAD) flow cytometry assay (BD Biosciences, San Jose, CA, USA) was used to define apoptosis and cell viability. The staining was performed according to manufacturer's instructions.

To assess the effect of g#5 on cell-cycle distribution, FACS analysis was performed on MM cells previously treated with gapmeRs after staining with Propidium Iodide (PI). At each time point, cells were collected, washed twice with phosphate-buffered saline (PBS) and fixed in cold 70% ethanol at 20°C. Before FACS analysis, cells were washed with PBS and stained in 50 µg/ml PI, 100 µg/ml RNase, 0.05% Nonidet P-40 for 1h at room temperature in the dark. Cell cycle profiles were determined using FCS Express 6 software.

Expression plasmids

pCDNA3.1 Flag-NRF2 (#36971) and Flag-NRF1 (#34707) plasmids were from Addgene (Cambridge, MA, USA). pEZ-M06 and pEZ-M06-EZH2 plasmids were from Genecopoeia (GeneCopeia, Rockville, MD, USA). MALAT1 cDNA in pCMV-SPORT6 (Dharmacon GE) was subcloned by the Genomics facility of Biogem IRGS (Ariano Irpino, Italy) using PmeI and BamH1 sites, in a pGREENpuro lentiviral vector (System Biosciences) where the H1 was replaced with CMV promoter. The plasmid containing MALAT1 promoter (2520bp, 2000bp upstream to 519bp downstream of the TSS) cloned in pEZX-PG04 vector upstream the luciferase gene was from Genecopoeia (catalogue# CS-HPRM25776-PG04).

Lentivirus production, transduction and transfection of MM cells

To obtain a lentivirus carrying MALAT1, 293Ta cells co-transfected using the calcium-phosphate technique with 10 µg of pGREENpuro-MALAT1 (or the pGREENpuro empty vector), 10 µg of pCMV-VSVG, and 4 µg of Δ8.9 plasmid. The supernatant was collected 48hours after 293Ta transfection and used to transduce MM cells by one round of infection

in the presence of 8 μg/mL of polybrene (Sigma-Aldrich, Saint Louis, Missouri). MM cells were then selected in medium containing 0.5 μg/ml puromycin.

To obtain MM cells stably expressing luciferase transgene, AMO-BZB cells were infected with pLenti-III-PGK-Luc Control Vector according to manufacturer's instruction(Applied Biological Materials Inc. Richmond, BC, Canada, Cat. #LV088), and two days after transduction, selection with 0.5 μg/mL of puromycin was performed for 3 days.

For transient expression, MM cells were electroporated by the Neon® Transfection System (Invitrogen, Life Technologies, Carlsbad, CA, USA) with the following protocol: 2 pulses, 1050 V, 30 milliseconds.

RIP

Cells (1.0 x10^7) were washed with cold PBS and lysed on ice for 15' using the harsh lysis buffer; supernatants were collected by centrifugation at 14.000 rpm/4°C, and then equally divided for O/N immunoprecipitation with KMT6/EZH2 antibody (Abcam ab3748) or rabbit IgG (Santa Cruz Biotechnology). IPs were incubated on a rotator with 20 μl of Protein A Magnetic Beads at 4°C. Bound magnetic beads were harvested and washed 5 times. Antibody-RNA binding protein complex was collected for RNA purification.

Quantitative real-time amplification (qRT-PCR)

The single-tube TaqMan assays (Applied Biosystems, Carlsbad, CA) used were: MALAT1 (Hs00273907_s1), PSMA2 (Hs00746751_s1), PSMA4 (Hs00160631_m1), PSMA5 (Hs00936004_m1), PSMβ4 (Hs01123843_g1), PSMβ5 (Hs00605652_m1), Rpn1 (Hs00161446), NFE2L1/NRF1 (Hs00231457_m1), NFE2L2/NRF2 (Hs00232352_m1), PSMβ1 (Hs00427351_m1), PSMβ6 (Hs00382586_m1), KEAP1 (Hs00202227_m1). GAPDH TaqMan assay Hs02786624_g1 was used for normalization. MALAT1 was also detected by SYBR Green qRT-PCR using the following primers: Fw, 5'-GAATTGCGTCATTTAAAGCCTAGTT -3'; Rev, 5' -GTTTCATCCTACCACTCCCAATTAAT -3' , and normalization performed with GAPDH: Fw, 5′-GAGTCAACGGATTTGGTCGT-3′, Rev: 5′-GACAAGCTTCCCGTTCTCAG-3′.

ChIP

Cells (1.5 x10^7) were crosslinked in 1% formaldehyde, lysed and sheared by sonication for 10 cycles (each of 30 seconds) on a cold block with 90 seconds time intervals of cooling

using the Bioruptor Plus (Diagenode). Chromatin was divided into equal amounts of immunoprecipitation with the histone H3 (tri methyl K27) antibody (ab6002), NRF1 (ab34682), from Abcam (Cambridge, MA, USA), or rabbit IgG as negative control (Santa Cruz Biotechnology). Chromatin extracts were incubated on a rotator with 20 μl of ChIP Grade Protein A/G Plus Agarose for 3 h at 4°C. Bound agarose beads were harvested by centrifugation (12.000 rpm, 15 seconds) and washed; the precipitated protein-DNA complexes were eluted from washed beads and incubated twice at 65°C for 1.5 h with NaCl and Proteinase K to revert cross-links.

Purified DNA was subjected to qPCR using GoTaq qPCR Master Mix (Promega). Primer sequences for qPCR were:

MALAT1 promoter:
Fw, 5' AGTAGCGACCGAGAAGTTCC;
Rev, 5' TGGTTCTAACCGGCTCTAGC.

KEAP1 promoter:
Fw, 5' TTTTCCCTAGATCCTGCGGC;
Rev, 5' CCTTCTCACTGTCCCTTCCATC.

Results are expressed as fold change as compared to IgG set as 1.

References

1. Ronchetti D, Agnelli L, Taiana E, Galletti S, Manzoni M, Todoerti K, et al. Distinct lncRNA transcriptional fingerprints characterize progressive stages of multiple myeloma. Oncotarget. 2016;7(12):14814-30.

2. Gulla A, Di Martino MT, Gallo Cantafio ME, Morelli E, Amodio N, Botta C, et al. A 13 mer LNA-i-miR-221 Inhibitor Restores Drug Sensitivity in Melphalan-Refractory Multiple Myeloma Cells. Clin Cancer Res. 2016;22(5):1222-33.
