# Supplementary material for: Drugging the lncRNA MALAT1 via LNA gapmeR ASO inhibits gene expression of proteasome subunits and triggers anti-multiple myeloma activity
Source: Leukemia. 2018 Feb 22;32(9):1948–57. doi: 10.1038/s41375-018-0067-3 (PMC6127082; doi:10.1038/s41375-018-0067-3)
Supplement: Supplementary file 2 — Supplementary Figures and Tables [file 41375_2018_67_MOESM2_ESM.pdf]

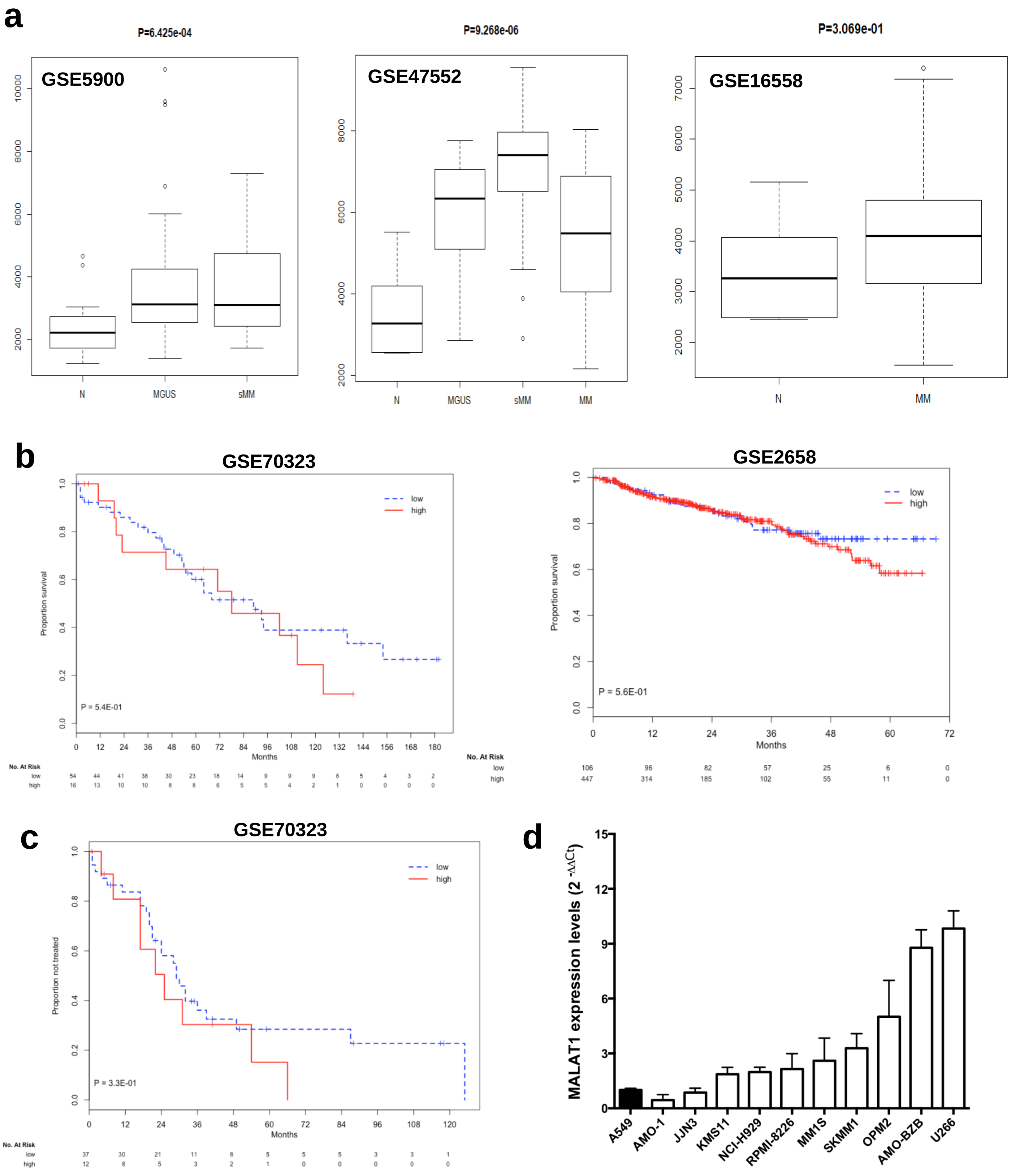
a
P=6.425e-04
GSE5900
N
MGUS
sMM
P=9.268e-06
GSE47552
N
MGUS
sMM
MM
P=3.069e-01
GSE16558
N
MM
b
GSE70323
Proportion survival
low
high
P = 5.4E-01
Months
No. At Risk
low 54 44 41 38 30 23 18 14 9 9 9 8 5 4 3 2
high 16 13 10 10 8 8 6 5 5 4 2 1 0 0 0 0
GSE2658
Proportion survival
low
high
P = 5.6E-01
Months
No. At Risk
low 106 96 82 57 25 6 0
high 447 314 185 102 55 11 0
c
GSE70323
Proportion not treated
low
high
P = 3.3E-01
Months
No. At Risk
low 37 30 21 11 8 5 5 5 3 3 1
high 12 8 5 3 2 1 0 0 0 0 0
d
MALAT1 expression levels (2 -ΔΔCt)
A549
AMO-1
JJN3
KMS11
NCI-H929
RPMI-8226
MM1S
SKMM1
OPM2
AMO-BZB
U266

Figure S1

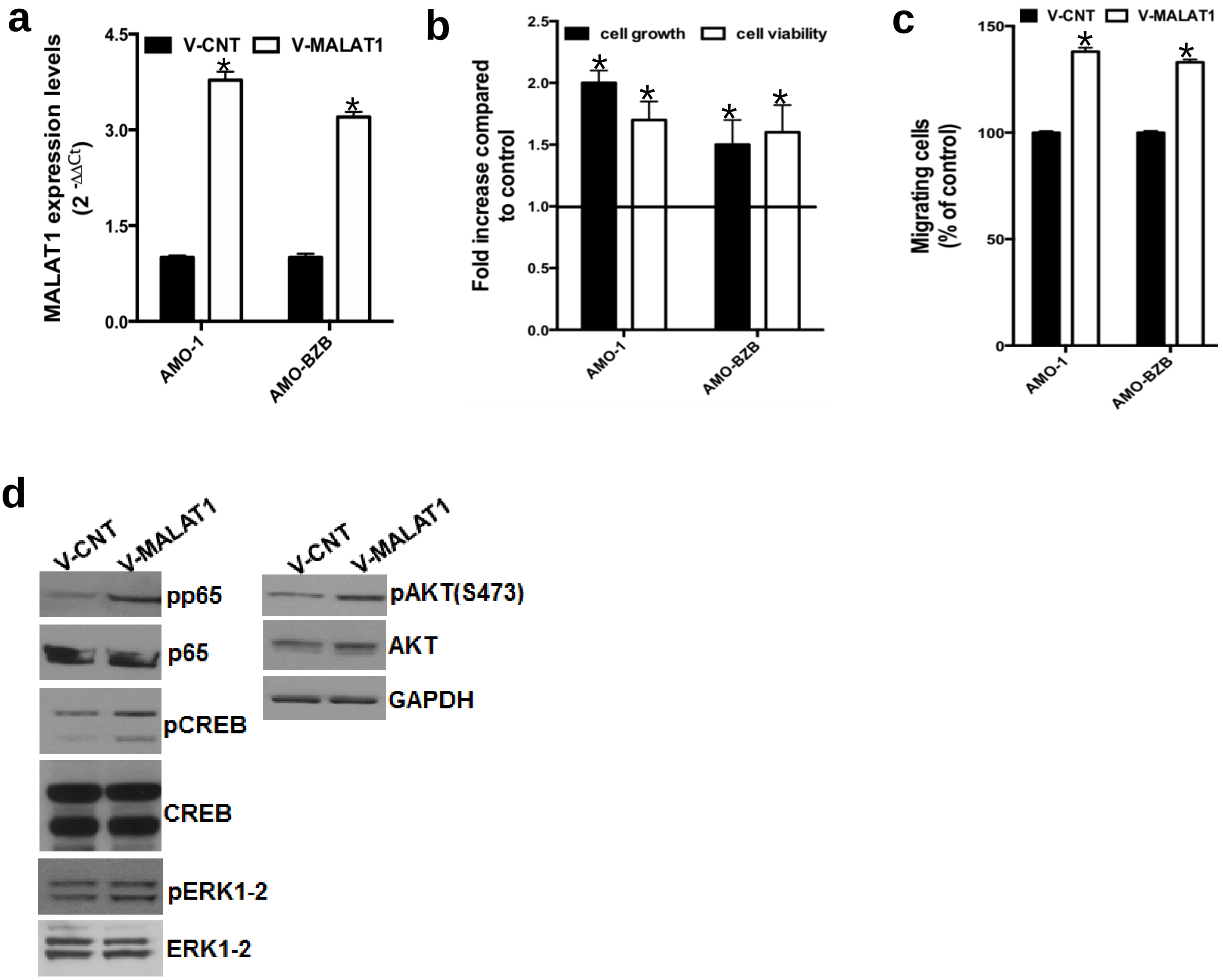
a
MALAT1 expression levels (2 -ΔΔCt)
V-CNT
V-MALAT1
AMO-1
AMO-BZB
b
Fold increase compared to control
cell growth
cell viability
AMO-1
AMO-BZB
c
Migrating cells (% of control)
V-CNT
V-MALAT1
AMO-1
AMO-BZB
d
V-CNT
V-MALAT1
pp65
p65
pCREB
CREB
pERK1-2
ERK1-2
pAKT(S473)
AKT
GAPDH

Figure S2

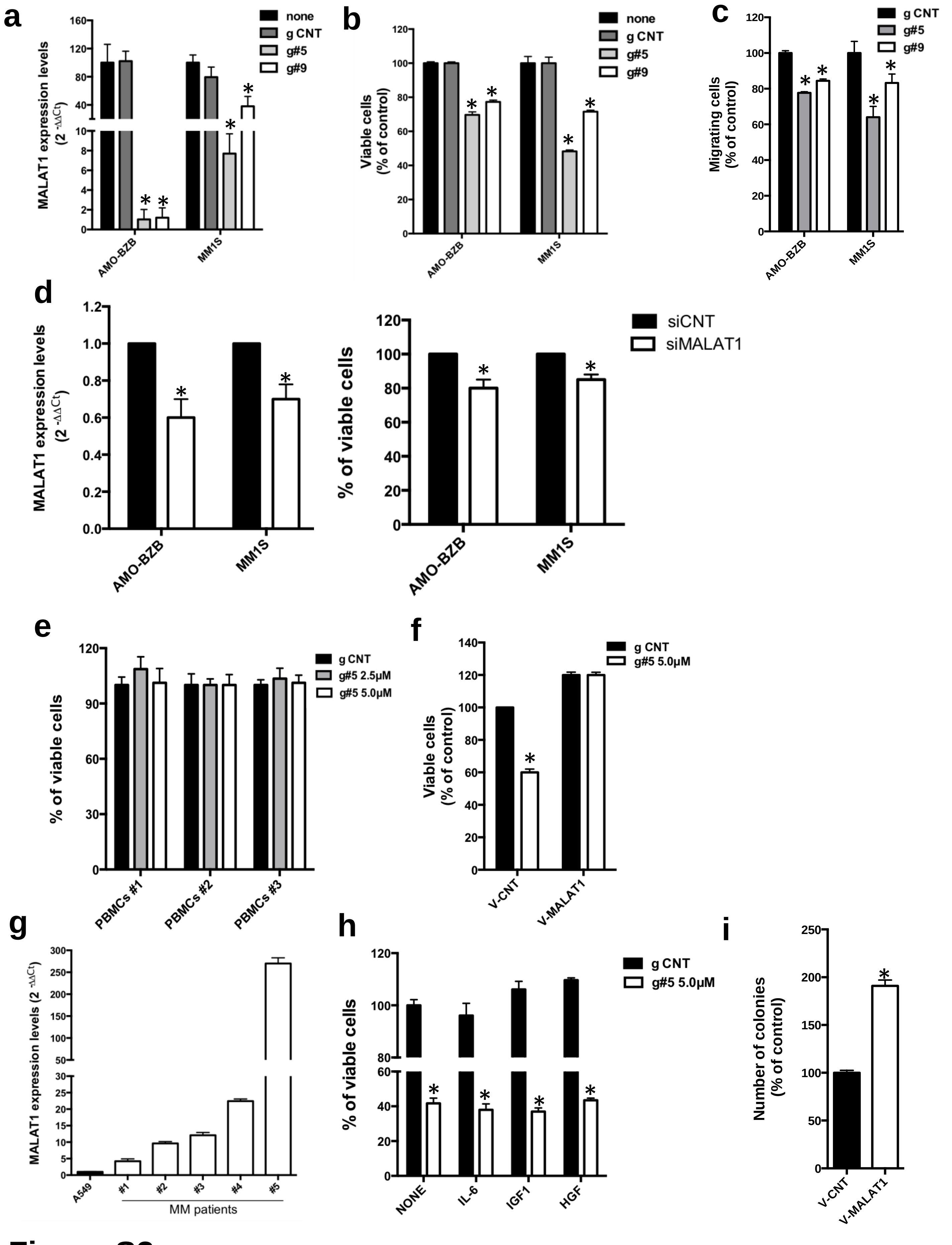
a
b
c
d
e
f
g
h
i
MALAT1 expression levels
Viable cells (% of control)
Migrating cells (% of control)
% of viable cells
Number of colonies (% of control)
none
g CNT
g#5
g#9
siCNT
siMALAT1
g#5 2.5μM
g#5 5.0μM
AMO-BZB
MM1S
PBMCs #1
PBMCs #2
PBMCs #3
V-CNT
V-MALAT1
A549
#1
#2
#3
#4
#5
MM patients
NONE
IL-6
IGF1
HGF

Figure S3

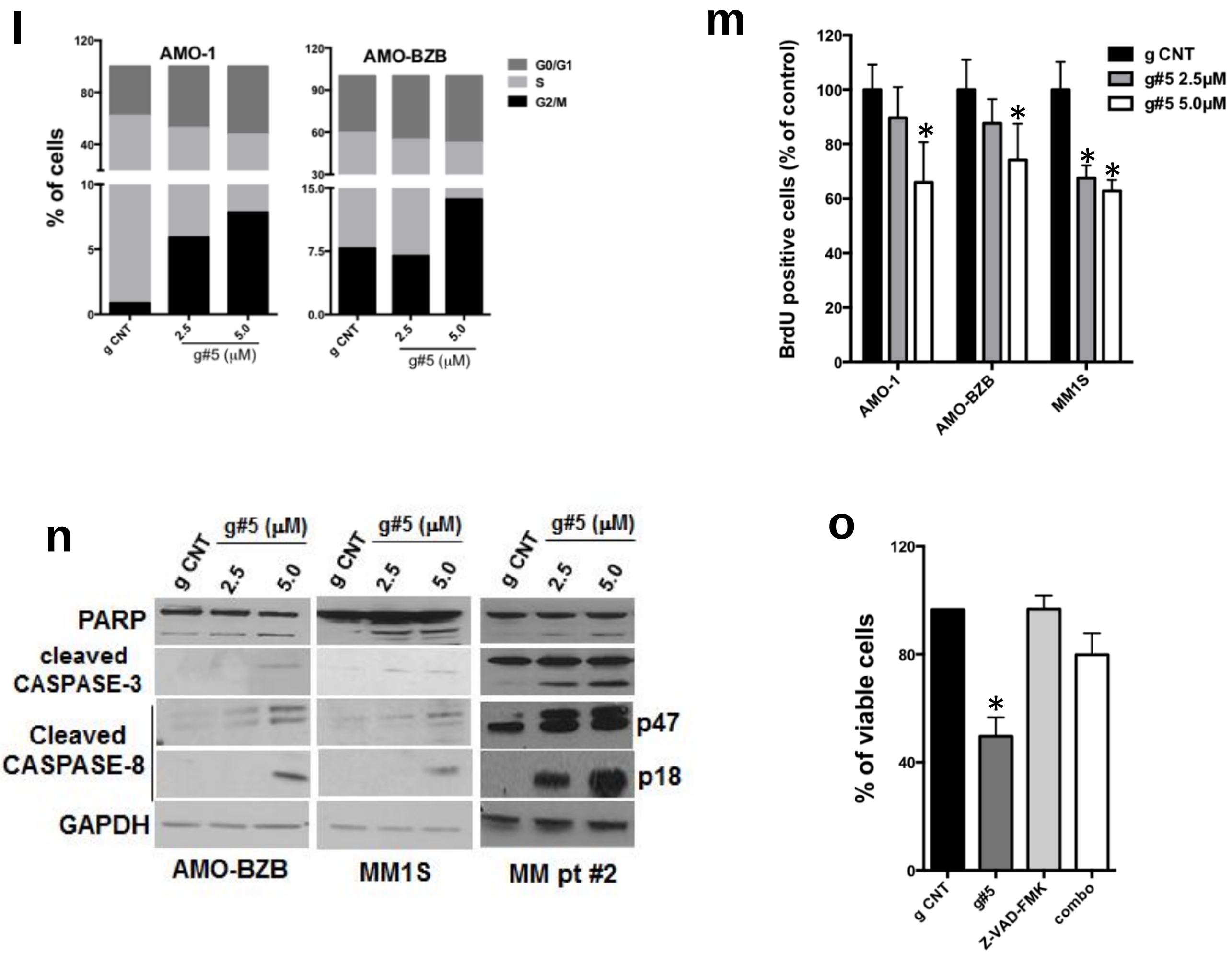

l
AMO-1
AMO-BZB
% of cells
G0/G1
S
G2/M
g CNT
2.5
5.0
g#5 (μM)
m
BrdU positive cells (% of control)
g CNT
g#5 2.5μM
g#5 5.0μM
AMO-1
AMO-BZB
MM1S
n
g CNT
g#5 (μM)
PARP
cleaved CASPASE-3
Cleaved CASPASE-8
p47
p18
GAPDH
AMO-BZB
MM1S
MM pt #2
o
% of viable cells
g CNT
g#5
Z-VAD-FMK
combo

FIGURE S3

Figure S4

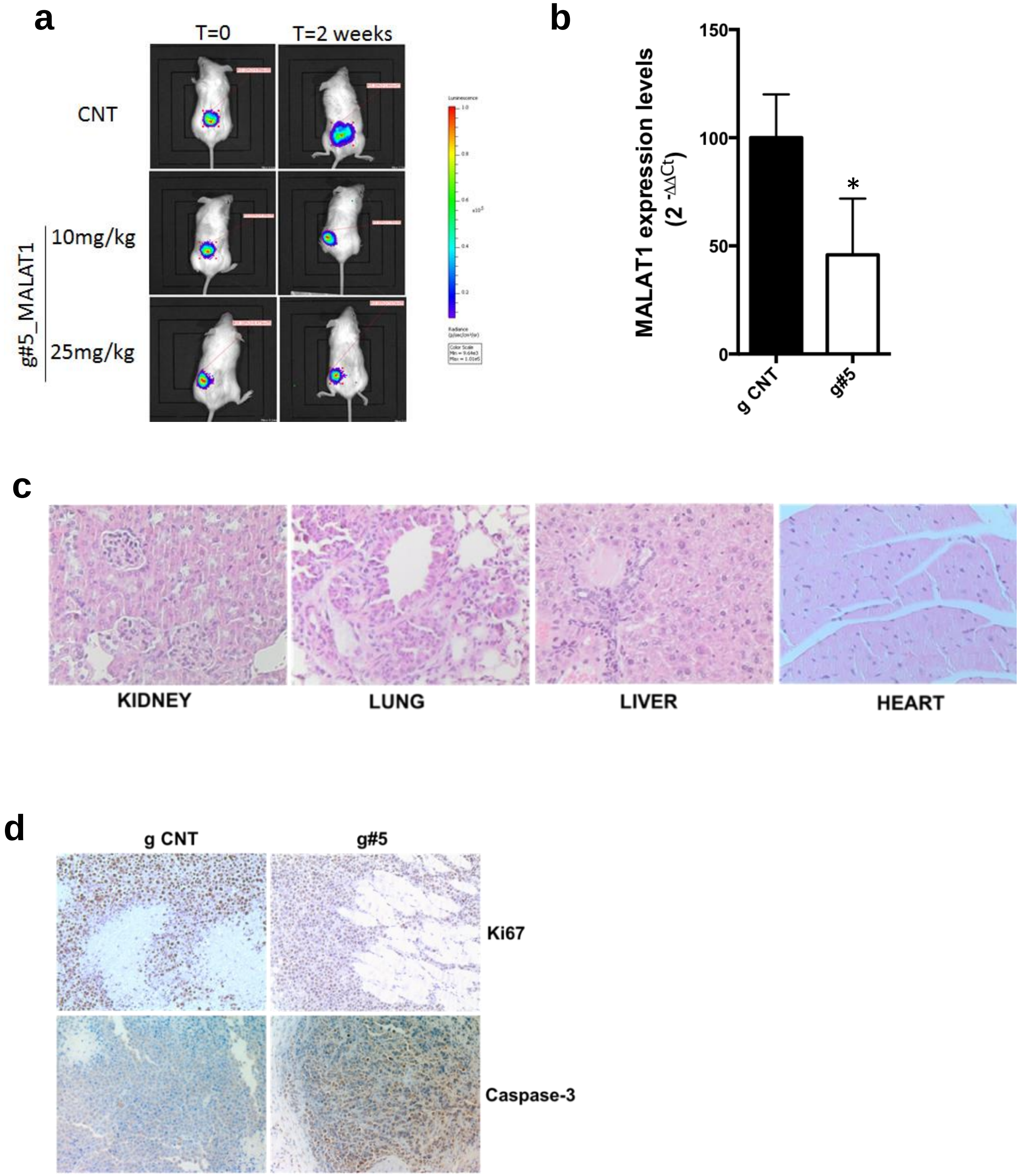
a
T=0
T=2 weeks
CNT
g#5_MALAT1
10mg/kg
25mg/kg
b
MALAT1 expression levels
(2 -ΔΔCt)
150
100
50
0
*
g CNT
g#5
c
KIDNEY
LUNG
LIVER
HEART
d
g CNT
g#5
Ki67
Caspase-3

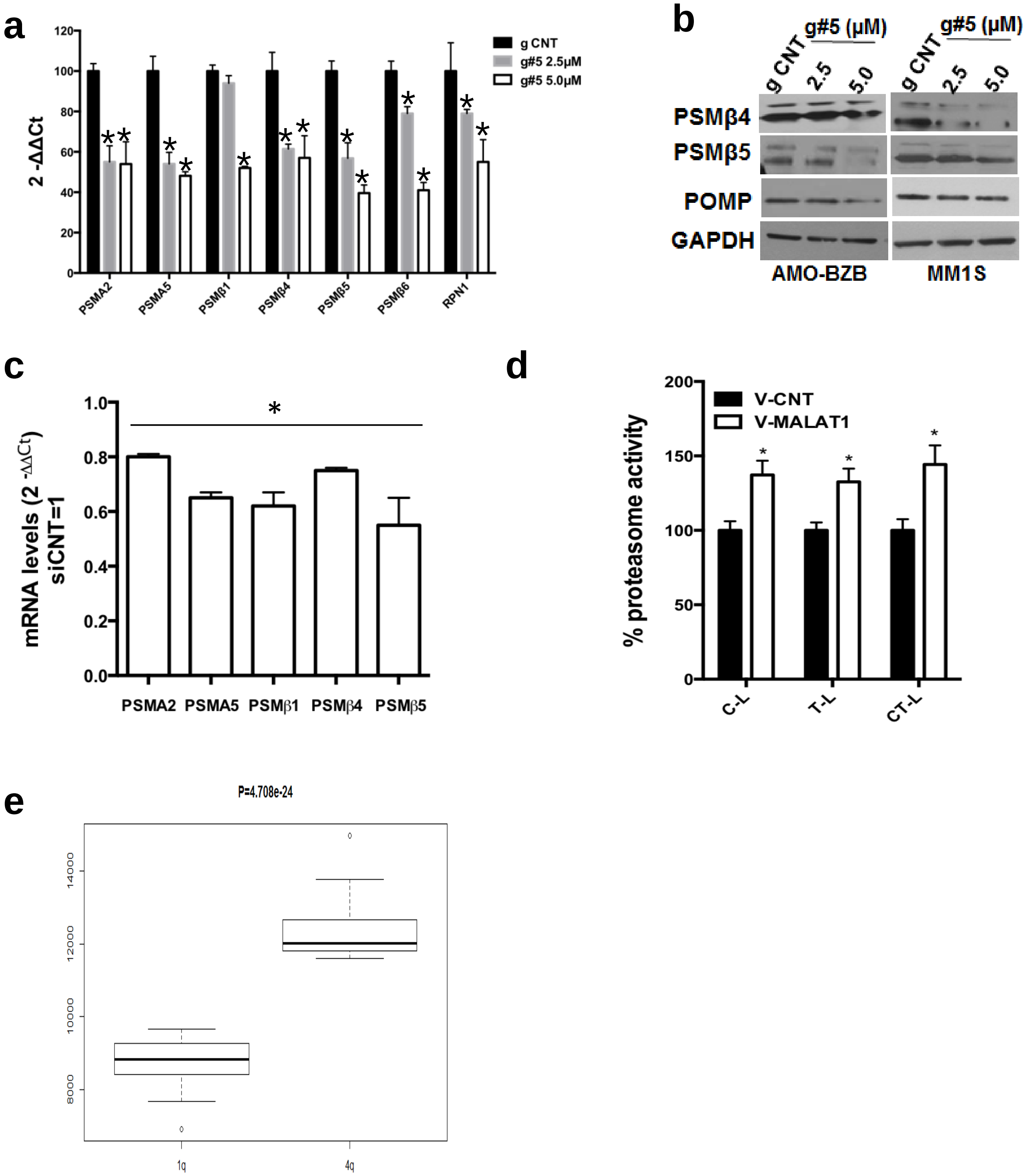
a
g CNT
g#5 2.5µM
g#5 5.0µM
2 -ΔΔCt
PSMA2
PSMA5
PSMβ1
PSMβ4
PSMβ5
PSMβ6
RPN1
b
g#5 (µM)
g CNT
2.5
5.0
PSMβ4
PSMβ5
POMP
GAPDH
AMO-BZB
MM1S
c
mRNA levels (2 -ΔΔCt) siCNT=1
PSMA2 PSMA5 PSMβ1 PSMβ4 PSMβ5
d
V-CNT
V-MALAT1
% proteasome activity
C-L
T-L
CT-L
e
P=4.708e-24
1q
4q

Figure S5

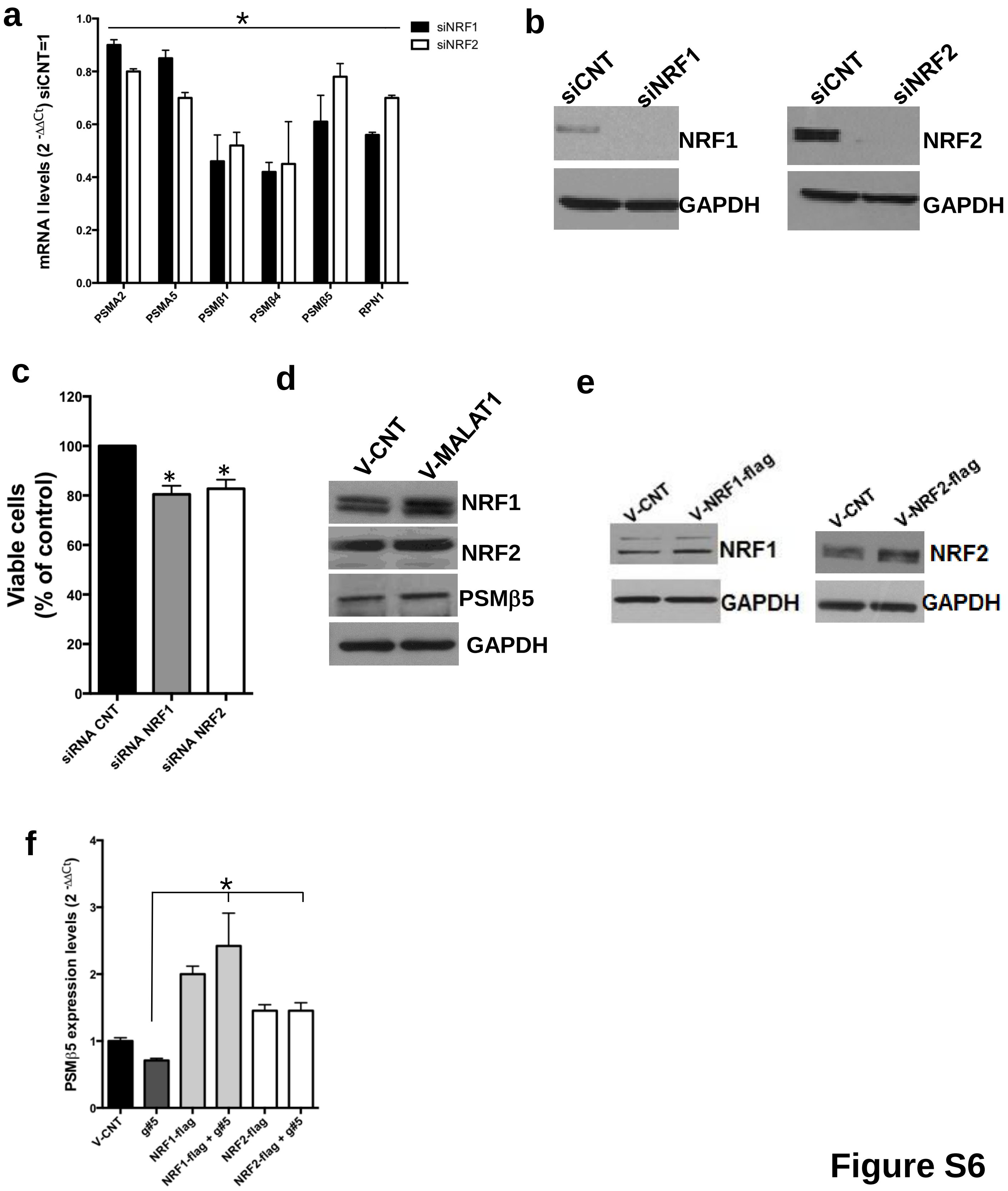
a
*
siNRF1
siNRF2
mRNA l levels (2 -ΔΔCt) siCNT=1
1.0
0.8
0.6
0.4
0.2
0.0
PSMA2
PSMA5
PSMβ1
PSMβ4
PSMβ5
RPN1
b
siCNT
siNRF1
NRF1
GAPDH
siCNT
siNRF2
NRF2
GAPDH
c
Viable cells (% of control)
120
100
80
60
40
20
0
*
*
siRNA CNT
siRNA NRF1
siRNA NRF2
d
V-CNT
V-MALAT1
NRF1
NRF2
PSMβ5
GAPDH
e
V-CNT
V-NRF1-flag
NRF1
GAPDH
V-CNT
V-NRF2-flag
NRF2
GAPDH
f
PSMβ5 expression levels (2 -ΔΔCt)
4
3
2
1
0
*
V-CNT
g#5
NRF1-flag
NRF1-flag + g#5
NRF2-flag
NRF2-flag + g#5

Figure S6

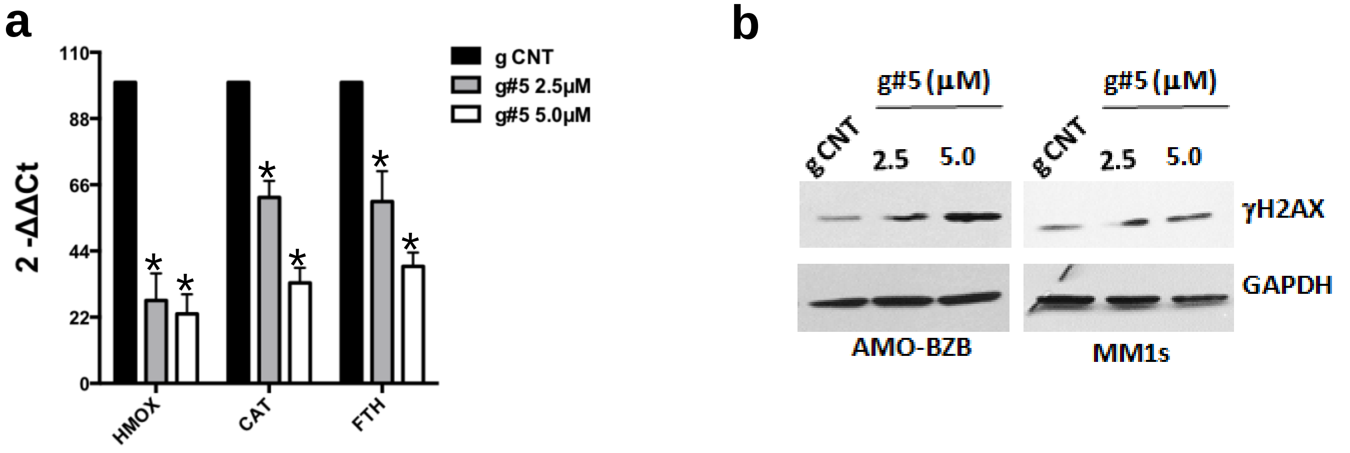

a
110
88
66
44
22
0
2 -ΔΔCt
g CNT
g#5 2.5μM
g#5 5.0μM
HMOX
CAT
FTH
b
g#5 (μM)
g CNT
2.5
5.0
γH2AX
GAPDH
AMO-BZB
MM1s

Figure S7

Figure S8

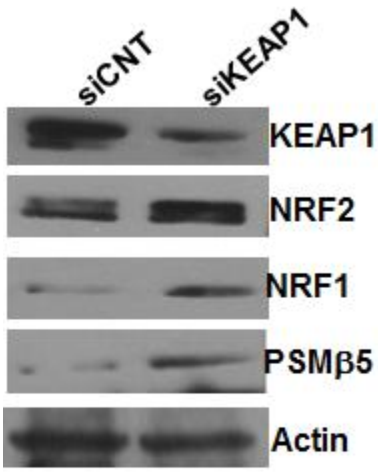

Figure S9

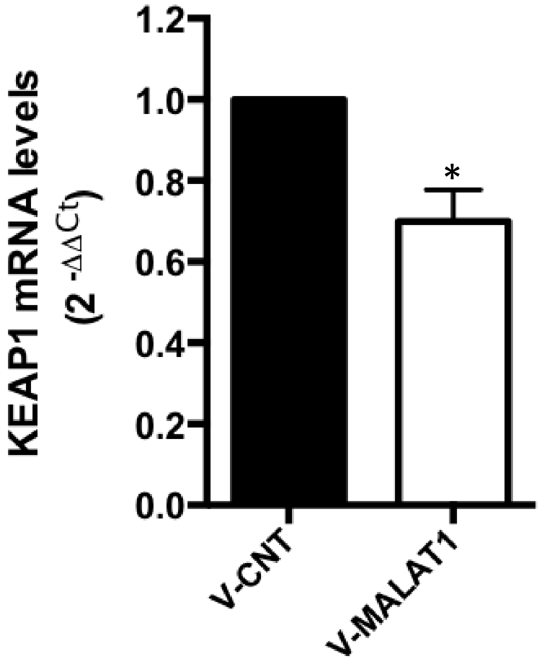

Figure S10

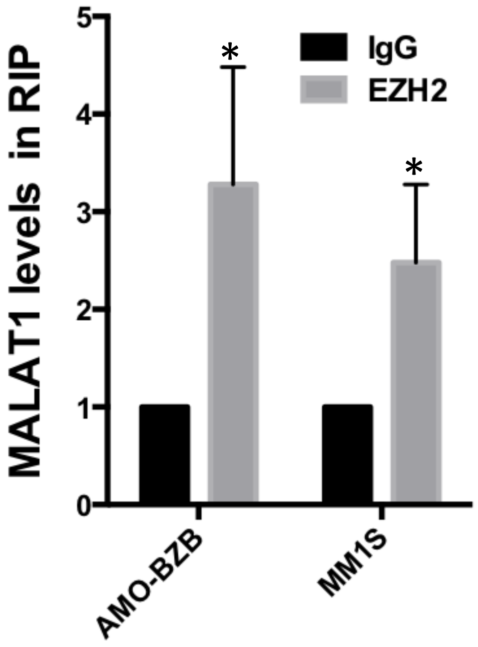

Figure S11

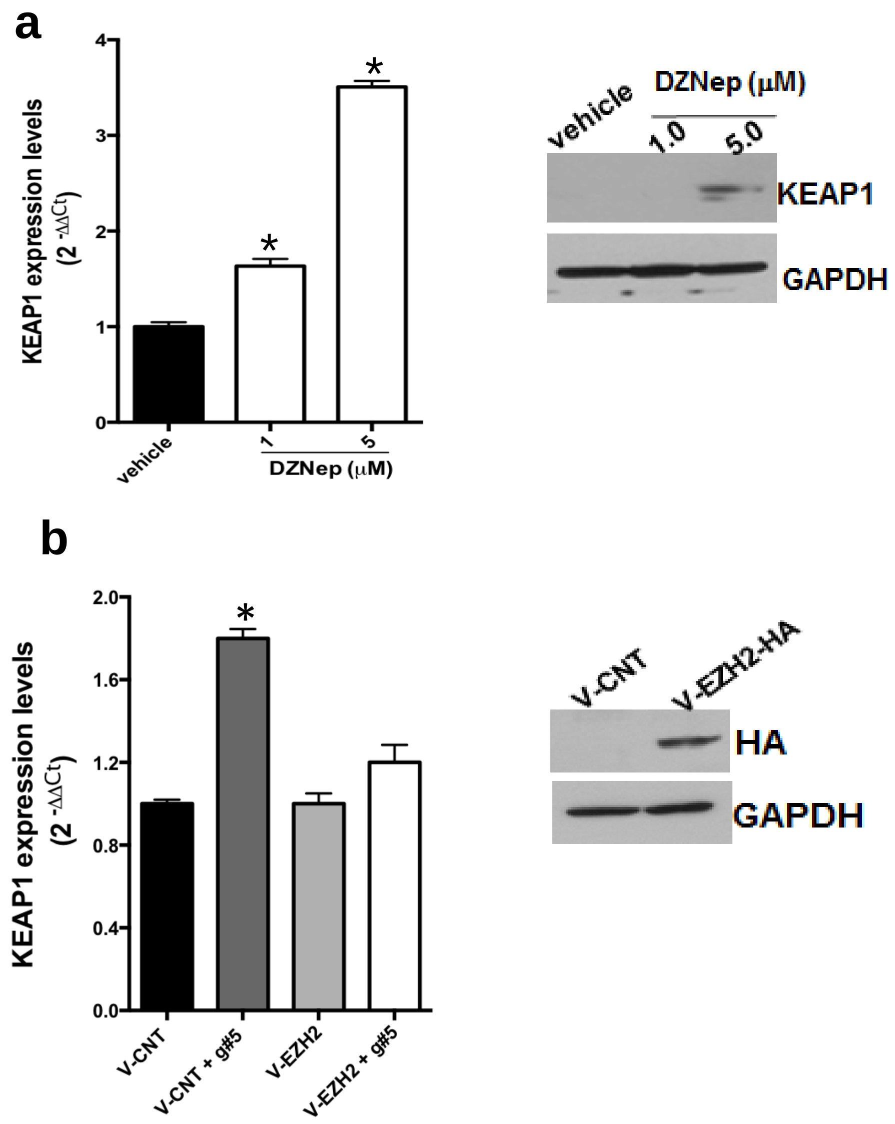
a
KEAP1 expression levels
(2 -ΔΔCt)
4
3
2
1
0
*
*
vehicle
1
5
DZNep (μM)
vehicle
DZNep (μM)
1.0
5.0
KEAP1
GAPDH
b
KEAP1 expression levels
(2 -ΔΔCt)
2.0
1.6
1.2
0.8
0.4
0.0
*
V-CNT
V-CNT + g#5
V-EZH2
V-EZH2 + g#5
V-CNT
V-EZH2-HA
HA
GAPDH

ATTATTTACGTTGTATTAGGTATTATAAGCAAACTAGAGATGATTTAAGGGATATGGGAG

GATATCTGTAGGTTACATAAGGAACTTGAGCATCTTCAGATTTTAGCATCCGAGGAGAGG

TTTTAGAACCAAGGCCCCATGGATACCAAGGGATGACTGTACAACATTCTGGCGGCTGGG

CACAGTGGCTCAAGCCTGTAATCCCAGCACTTTGGGAGGCCGAGG**C**GGGCGGATCACGAG

GTCAGGAGATCCAGACCATCCTGGTTAACACGGTGAAACCCTGTCTCTGCTAAAACTACA

AAAAATTAGTCGGGCGTGGTGGCGGGCGCCTGTAGTACCAGCTACTCGGGAGGCTGAGGC

AAGTGAATTGCTTGAACCAGGGAGGCGGAGGTTACGGTGAGCCGAGATCCCGCCACTGCA

CTCCAGCCTCAGCGATAGAGTGAGACTTCGTCTCAAAAAAAAAAAAAAAGAAACATTCTG

TAATCTGTCAAGGTAATGGAAATTAATGCAGTGACACTGACAATAATTTAAAGCATTTTA

GGGCTCTTAACGTTCATCTCACTCGCTCTAGAGTGAAGGAGAGAGCAGAGAGTACAGAGC

AGGTCCTAGGTCCTCCCCCAGATATAGAAAAACCGCAGTTGGAGAGACTGACAGAAGTGC

TGGAGAGGAAGGGAGGCAGCAGGACGTGGAAAATCTGGGATTCAAGAACAGCTTCTGCTA

TTAATTAGCTGTGTGTCATTTCAGGCAAATCACAAAATCTCCAGGCCCCCAAATCCCCAT

TTGCGAAACGAGGTGACCTGCCTCAGTTTTCCTTCATCCAGCGATAAAATTCTACTAAAA

AATTTCCAACCCCAGAGCAAGCATCAGGACTGTGCCTGAAGGGATCCGGCTGCATCTCAG

TAATATTCCACTCTTACAGGCTTAAAAATAAAAAAGAAAAGAAAAGAAAACAAGAACAGT

TTCACCAGCGTCAATTGAGAACAGCTGCTTCAACAGGCCCTGCTTTATGTGGGCAGGTGG

GGACAGGGAGTTGGCCCAGAAAACAGGACCCTCATTTCCTGGAGCCCCCAGGGCAGCTCC

CCAACACCGTACACAACCTGCATCATTTACAGGAGCCAAAGGAGTTTTAGAAATCAACTT

CATAGAGTTGCTGTATCATTGTGTTAGTTTTGCCATCCTAACCTATACAGCGTCACTAAT

CTCTCCCCTCGGAGTTGACTGCCTAAAAACAAGCCATGGATACAGGTTCCAAAGACCCGG

GGGTGAGGGAGCGGAGAGGGTGGGTGTCACCACCCCGTCCAGCTCCAAGCTTTGTGTGCC

CTGGAACTCTCCATTTTAGGTCATTGCTTCAGTTTCTTTTCTAAAAAATTAGGCTGCTGC

AAGGTCAGCCTGAGACCACTTCTGCCCCGAGAATTCTAGACTAGTAAGACCTGGTGACAT

ACAAACGACGAAGATATTTTACAATAATGCCATGGCCCTTGATAGCTACACGAGGTTTGT

GTTCTGATTTTAAATTAATGGATGACGTCGAGACTATATGGAGGAAATGACAAAGGACAG

GAGAGAGGTGGGAAAGGAAGACCTAGACTGAAAATGGAAGTTGGGCAGCAGCTCCACGAA

AGAAAGACCAGCCCCCAAGTGCAGTGACAGCGCAG**AGTAGCGACCGAGAAGTTCC**CAGGC

CAGCTGCCACCCCGCCCCCATGCCATTCCCCAGAACAGGCACAGGCGTTAGGGCGGGGCG

CGCGTGCGCAGTCACGCGCTGCGCCAACCGCCACAGCTCCGGGAAGGCGGCCAGGACCG**G**

CTAGAGCCGGTTAGAACCAGTGGCGCCCGCCCACGAGCCAGCGCCTCACAAAGGGAGGGC

GGCTCACGGCCCTCGCGTATCCCTGCGCGGCGCTCGCGAGCCGCCCCTCCCCCGGCGTTT

GTCCCTGACGCAGCCCCACCGGTTGCGCAGTCCCTCCCCGCCCCCGCTCTCCCCTCCGCA

GCCTGCAGCCCGAGACTTCT**GTA**AAGGACTGGGGCCCCGCAACTGGCCTCTCCTGCCCTC

TTAAGCGCAGCGCCATTTTAGCAACGCAGAAGCCCGGCGCCGGGAAGCCTCAGCTCGCCT

GAAGGCAGGTCCCCTCTGACGCCTCCGGGAGCCCAGGTTTCCCAGAGTCCTTGGGACGCA

GCGACGAGTTGTGCTGCTATCTTAGCTGTCCTTATAGGCTGGCCATTCCAGGTGGTGGTA

TTTAGATAAAACCACTCAAACTCTGCAGTTTGGTCTTGGGGTTTGGAGGAAAGCTTTTAT

TTTTCTTCCTGCTCCGGTTCAGAAGGTCTGAAGCTCATACCTAACCAGGCATAACACAGA

ATCTGCAAAACAAAAACCCCTAAAAAAGCAGACCCAGAGCAGTGTAAACACTTCTGGGTG

TGTCCCTGACTGGCTGCCCAAGGTCTCTGTGTCTTCGGAGACAAAGCCATTCGCTTAGTT

GGTCTACTTTAAAAGGCCACTTGAACTCGCTTTCCATGGCGATTTGCCTTGTGAGCACTT

Figure S12

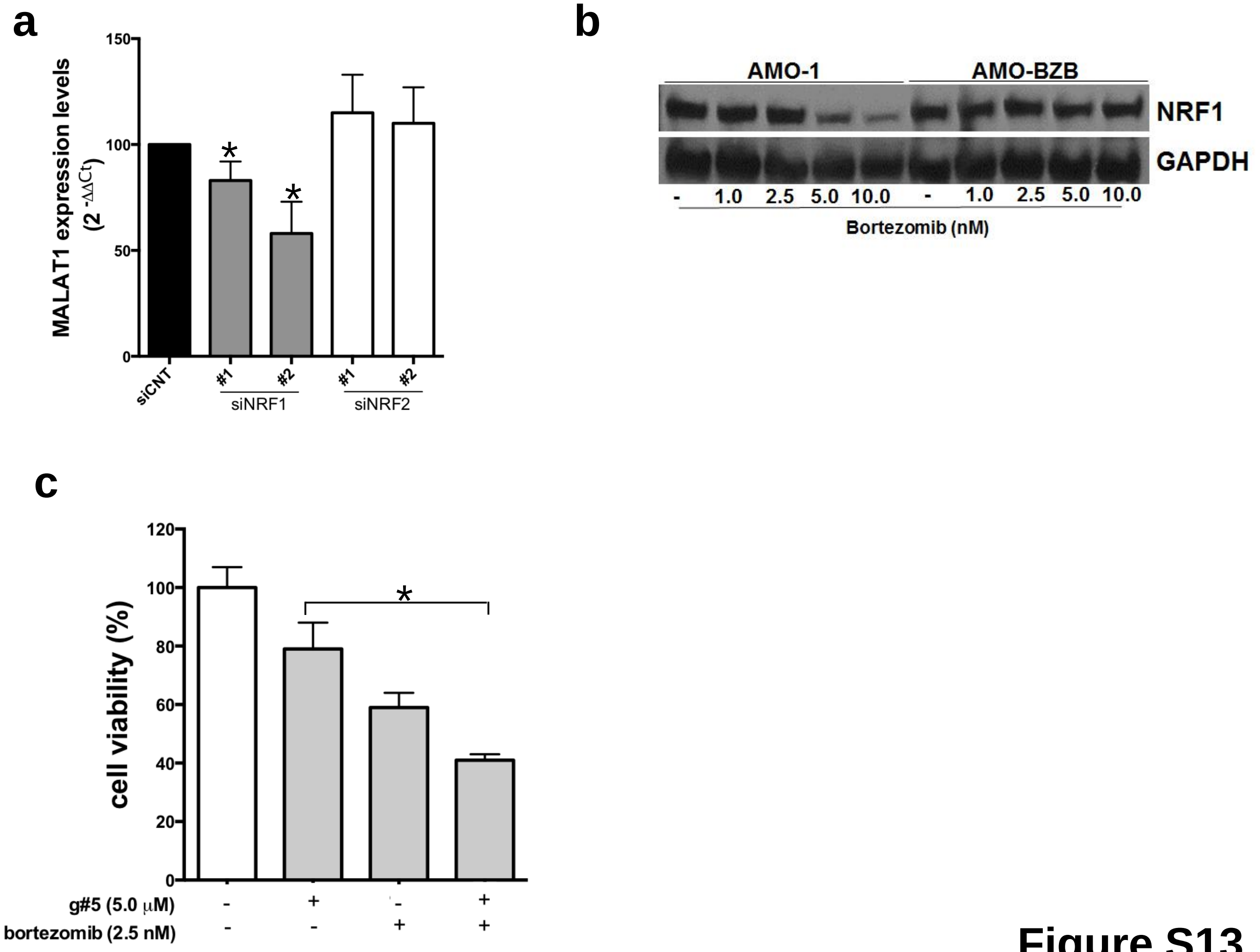
a
MALAT1 expression levels
(2 -ΔΔCt)
150
100
50
0
*
*
siCNT
#1
#2
siNRF1
#1
#2
siNRF2
b
AMO-1
AMO-BZB
NRF1
GAPDH
- 1.0 2.5 5.0 10.0 - 1.0 2.5 5.0 10.0
Bortezomib (nM)
c
cell viability (%)
120
100
80
60
40
20
0
*
g#5 (5.0 μM) - + - +
bortezomib (2.5 nM) - - + +

Figure S13

SUPPLEMENTARY TABLE S1

List of the significantly enriched functional annotation clusters for the 1093 differentially expressed genes, by DAVID Bioinformatics Resources 6.8.

Category	Term	Count	PValue	Genes
Annotation Cluster 1	Enrichment Score: 3.001856655769477			
GOTERM_BP_DIRECT	GO:0006412~translation	28	3.26E-04	RPL17, CPEB2, MRPS14, COPS5, MRPS11, RPL36, AKT1, RPL6, RPL8, MRPL37, RPL11, RSL24D1, SLC25A26, TNIP1, SLC25A40, RPSA, MRPL51, SLC25A6, MRPS24, MRPS21, MRPS7, SLC25A11, MRPL22, MRPS18C, MRPL28, MRPS18A, RPS14, MRPL43
Annotation Cluster 2	Enrichment Score: 2.554136764373698			
**GOTERM_CC_DIRECT**	**GO:0000502~proteasome complex**	**13**	**4.21E-05**	**UBE3A, PSMG3, PSMA2, PSMD14, PSMB6, PSMG2, PSMD12, PSME1, PSMC4, PSME2, PSMD10, PSMD6, PSMD8**
Annotation Cluster 3	Enrichment Score: 2.5151342665277356			
GOTERM_MF_DIRECT	GO:0061630~ubiquitin protein ligase activity	22	8.47E-04	RNF220, UBE3A, BTRC, CBL, MED12, SKP2, CDC34, TOPORS, RLIM, UBE2B, MED10, ZNRF2, PJA2, MED31, RNF6, MED17, RABGEF1, SIAH3, RNF138, UBE2W, RNF149, MED1
Annotation Cluster 4	Enrichment Score: 2.419882297050254			
GOTERM_MF_DIRECT	GO:0003924~GTPase activity	28	1.05E-04	GPN1, RAB3A, ATL2, RAB5C, GTPBP10, EIF5B, BMS1, RAB1A, TUBB, DDX3X, RALB, RRAS, TUBG1, TUBG2, RAB21, RAB27A, RAB2A, EEF1A1, GTPBP4, OPA1, ERAL1, TRIM23, RAB33B, MFN1, RAB18, RAB22A, RIT1, RAP1B
Annotation Cluster 5	Enrichment Score: 2.295220723001088			
GOTERM_BP_DIRECT	GO:0010467~gene expression	12	2.74E-05	HNRNPA3, POLR2H, HNRNPM, POLR2F, HNRNPK, POLR2K, PTBP1, HNRNPA2B1, HNRNPH1, HNRNPA1, POLR2B, HNRNPU
Annotation Cluster 6	Enrichment Score: 2.0231878347278887			
GOTERM_BP_DIRECT	GO:0032981~mitochondrial respiratory chain complex I assembly	10	0.005085069	NDUFV3, TIMMDC1, NDUFA5, NDUFB11, NDUFA2, NDUFB10, NDUFA6, BCS1L, NDUFA12, NDUFAF3

Table S1
