# Supplementary material for: Drugging the lncRNA MALAT1 via LNA gapmeR ASO inhibits gene expression of proteasome subunits and triggers anti-multiple myeloma activity
Source: Leukemia. 2018 Feb 22;32(9):1948–57. doi: 10.1038/s41375-018-0067-3 (PMC6127082; doi:10.1038/s41375-018-0067-3)
Supplement: Supplementary file 3 — Legends to Supplementary Figures and Tables [file 41375_2018_67_MOESM3_ESM.pdf]

Legends to Supplementary Figures

Figure S1. MALAT1 levels in MM primary samples and cell lines. a. MALAT1 relative expression levels in publicly available datasets. Log2 relative expression values are reported in y axis. Kaplan-Meier estimated curves for overall survival (**b**) and time to relapse (**c**) for MM patients' groups stratified according to k-means clustered MALAT1 expression level. **d**. MALAT1 relative expression levels were determined by qRT-PCR in a panel of MM cell lines (white columns). A549 non-small cell lung cancer (NSCLC) cell line (black column) was used as positive control for MALAT1 expression; data are representative of at least 3 independent experiments.

Figure S2. Effects of MALAT1 overexpression on MM cell viability, migration and signal transduction pathways. a. MALAT1 relative levels in cells transduced with empty (V-CNT) or MALAT1 (V-MALAT1) vectors. **b**. Cell growth and viability determined in V-CNT or V-MALAT1 transduced cells, by trypan blue and CTG assays respectively. **c**. Transwell migration assay was performed in V-CNT or V-MALAT1 transduced cells. **d.** WB of phospho-p65, p65-NF-KB, phospho-CREB and CREB, pERK1/2 and ERK1/2, pAKT(S473) and AKT, in MALAT1-overexpressing AMO1 cells. GAPDH was used as loading control. Blots from a representative experiments are shown.

Figure S3. *In vitro* effects of MALAT1 inhibition on MM cell lines and healthy PBMCs. MALAT1 levels (**a**) and cell viability (**b**) were determined after 48 hours by qRT-PCR and CTG assay respectively, in mock-electroporated (none) AMO-BZB and MM1S cells, or after electroporation with 50nM of g CNT or anti-MALAT1 LNA gapmeRs g#5 and g#9. **c**. Migration was assessed by transwell assay, 48 hours after transfection with 50nM g CNT, or anti-MALAT1 gapmeRs g#5 and g#9. **d.** MALAT1 levels (left panel) and cell viability (right panel) were determined after 48 hours by qRT-PCR and CTG assay respectively, in AMO-BZB and MM1S cells electroporated with 500nM of scramble siRNA (siCNT) or MALAT1 targeting siRNA pool (siMALAT1). **e**. Cell viability was assessed by CTG assay in PBMCs from three different healthy donors, 5 days after delivery of 2.5 or 5.0 μM naked g#5 or 5.0 μM g CNT. **f**. CTG assay performed in AMO-1 cells transduced with the empty vector (V-CNT) or MALAT1 (V-MALAT1) lentiviral vector, and then treated for 5 days with naked g CNT or g#5. **g**. MALAT1 relative expression levels were determined by qRT-PCR in purified primary PCs from 5 newly-diagnosed MM patients; A549 NSCLC cell line was used as positive control for MALAT1 expression. **h**. CTG cell viability assay was performed in AMO-BZB cells treated with 5.0 μM g #5 for 5 days, in the presence of absence of IL-6 (2.5 ng/ml), IGF-1 and HGF (both 100μg/ml). **i**. Methylcellulose colony formation assay was performed in AMO-1 cells transduced with empty vector (V-CNT) or MALAT1 vector (V-MALAT1). **l**. Cell cycle analysis by PI staining was performed in AMO-1 and AMO-BZB cells 72hours after treatment with g CNT or g#5. **m**. BrdU uptake was evaluated in MM cell lines, 72hours after treatment with naked g#5. **n.** WB of PARP, cleaved caspase 3 and 8 in MM cell lines or primary PCs, 5 days after delivery of naked g CNT or g#5. **o**. CTG assay was performed in AMO-BZB cells treated for 5 days with 5.0 g#5, with or without 25μM ZVAD-FMK. Data are representative of at least 3 independent experiments. * = $p<0.05$.

Figure S4. *In vivo* effects of g#5 on MM xenografts. a. Imaging analysis by IVIS-Lumina II system of AMO-BZB xenografts before and after g#5 treatment. **b**. MALAT1 relative expression levels were determined at day 21 in ABZB xenografts by qRT-PCR. **c**. H&E

staining of tissues from a representative mouse at day 21. **d**. IHC staining of caspase 3 and Ki67 on xenografts retrieved at day 21. * = $p<0.05$.

Figure S5. Manipulation of NRF1, NRF2 and MALAT1 levels affects proteasome gene expression and activities. a. qRT-PCR of indicated transcripts in MM1S cells, 4 days after delivery of naked gCNT (5.0 μM) or anti-MALAT1 g#5. **b**. WB of PSMβ4/5, POMP and GAPDH, 4 days after delivery of naked gCNT or anti-MALAT1 g#5. **c**. Relative mRNA expression of the indicated genes, 48 hours after electroporation of AMO-BZB with 500nM of scramble siRNA (siCNT) or MALAT1 targeting siRNA pool (siMALAT1). The expression of the indicated mRNAs after electroporation with 500nM of siRNA negative control (siCNT) was set as 1. **d**. Caspase-like (C-L), Trypsin-like (T-L) and Chymotrypsin like (CT-L) proteasome activities were determined by Proteasome-Glo assay (Promega) in AMO-1 cells transduced with the empty vector (V-CNT) or MALAT1 (V-MALAT1) lentiviral vector. Data are representative of at least 3 independent experiments. * = $p<0.05$. **e**. Box plot reporting MALAT1 levels in high (I quartile) and low (IV quartile) MALAT1 groups from GSE66293 dataset. Log2 relative expression values are reported on y axis.

Figure S6. *In vitro* effects of NRF1 and NRF2 in AMO-BZB cells. a. Relative mRNA expression of the indicated genes, 48 hours after transfection of 100nM siRNAs targeting NRF1, NRF2 or scrambled (siNC) control. WB analysis of NRF1 and NRF2 in AMO-BZB (**b**), and CTG cell viability assay (**c**), 48hours after transfection with scrambled oligos (siNC) or siRNAs targeting NRF1 (siNRF1) or NRF2 (siNRF2). **d**. WB analysis of NRF1, NRF2 and PSMβ5 levels in AMO-1 cells transduced with empty vector or MALAT1 lentiviral vector. **e**. WB of NRF1 and NRF2 protein, 48hours after transfection of NRF1 or NRF2 expression plasmids. **f**. qRT-PCR of PSMβ5 in AMO-BZB cells transfected with 2.5 μg of NRF1-flag or NRF2-flag expression vectors, and then treated for 4 days with 2.5μM naked g#5. Data are representative of at least 3 independent experiments. * = $p<0.05$.

Figure S7. Effects of g#5 on the expression of anti-oxidant genes and DNA damage. a. Relative mRNA levels of hemoxigenase-1 (HMOX), catalase (CAT) and ferritin heavy-chain (FTH) in AMO-BZB cells, 4 days after gymnotic delivery of g#5 or g CNT. **b**. WB of γH2AX, 4 days after delivery of g CNT (5.0 μM) or g#5.

Figure S8. Silencing of KEAP1 upregulates NRF1, NRF2 and PSMβ5 expression. WB analysis of KEAP1, NRF1, NRF2 and PSMβ5 proteins in AMO-BZB, 48hours after transfection with scrambled oligos (siNC) or siRNAs targeting KEAP1 (siKEAP1). Actin was used as loading control. Blots from a representative experiments are shown.

Figure S9. Overexpression of MALAT1 decreases KEAP1 mRNA levels. qRT-PCR of KEAP1 in AMO-1 cells transduced with the empty vector (V-CNT) or MALAT1 (V-MALAT1) lentiviral vector. Data are representative of at least 3 independent experiments. * = $p<0.05$.

Figure S10. EZH2 interacts with MALAT1 in AMO-BZB and MM1S cells. RIP assay was performed as indicated in Supplementary Methods, using an EZH2 antibody or IgG control. MALAT1 levels were determined by qRT-PCR after RIP. Data are representative of at least 3 independent experiments. * = $p<0.05$.

Figure S11. EZH2 enforced expression abrogates g#5-induced upregulation of KEAP1 mRNA. a. KEAP1 mRNA levels in AMO-BZB cells, 24hours after treatment with DZNep; WB of KEAP1 is reported on the right. **b.** AMO-BZB cells were transfected with 2.5 μg of EZH2 plasmid (V-EZH2-HA) or empty vector (V-CNT), and then treated for 4

days with 2.5μM naked g#5. KEAP1 relative mRNA expression was assessed by qRT-PCR; WB shows EZH2 levels in transfected cells. Data are representative of at least 3 independent experiments. * = $p<0.05$

Figure S12. Detection of an NRF1 binding site in MALAT1 promoter. A putative consensus corresponding to NRF1 binding site (highlighted in red) identified by TRANSFAC 7.0 database.

Figure S13. NRF1 regulates MALAT1 within a feedback loop affecting bortezomib sensitivity of MM cells. a. qRT-PCR of MALAT1 in AMO-BZB, 24hours after transfection with 100nM of NRF1 or NRF2 siRNAs. **b**. WB of NRF1, 24 hours after bortezomib treatment. **c**. CTG assay performed in primary PCs from a newly-diagnosed MM patient treated for 4 days with g#5 and bortezomib. Data are representative of at least 3 independent experiments. * = $p<0.05$

Supplementary Table S1. List of the significantly enriched functional annotation clusters for the 1093 differentially expressed genes, by DAVID Bioinformatics Resources 6.8. The proteasome pathway enriched in high MALAT1 patients is highlighted in red.
